# Spontaneous metastasis xenograft models link CD44 isoform 4 to angiogenesis, hypoxia, EMT and mitochondria‐related pathways in colorectal cancer

**DOI:** 10.1002/1878-0261.13535

**Published:** 2023-11-03

**Authors:** Arun Everest‐Dass, Stepan Nersisyan, Hanna Maar, Victor Novosad, Jennifer Schröder‐Schwarz, Vera Freytag, Johanna L. Stuke, Mia C. Beine, Alina Schiecke, Marie‐Therese Haider, Malte Kriegs, Omar Elakad, Hanibal Bohnenberger, Lena‐Christin Conradi, Maria Raygorodskaya, Linda Krause, Mark von Itzstein, Alexander Tonevitsky, Udo Schumacher, Diana Maltseva, Daniel Wicklein, Tobias Lange

**Affiliations:** ^1^ Institute for Glycomics Griffith University, Gold Coast Campus Australia; ^2^ Faculty of Biology and Biotechnology HSE University Moscow Russia; ^3^ Institute of Molecular Biology The National Academy of Sciences of the Republic of Armenia Yerevan Armenia; ^4^ Armenian Bioinformatics Institute (ABI) Yerevan Armenia; ^5^ Institute of Anatomy and Experimental Morphology University Medical Center Hamburg‐Eppendorf Germany; ^6^ Shemyakin‐Ovchinnikov Institute of Bioorganic Chemistry Russian Academy of Sciences Moscow Russia; ^7^ Department of Radiobiology and Radiation Oncology University Medical Center Hamburg‐Eppendorf Germany; ^8^ Institute of Pathology University Medical Center Göttingen Germany; ^9^ Clinic for General, Visceral and Pediatric Surgery University Medical Center Göttingen Germany; ^10^ Institute of Medical Biometry and Epidemiology University Medical Center Hamburg‐Eppendorf Germany; ^11^ Art Photonics GmbH Berlin Germany; ^12^ Medical School Berlin Germany; ^13^ Department of Anatomy and Cell Biology University of Marburg Germany; ^14^ Institute of Anatomy I Jena University Hospital Germany; ^15^ Comprehensive Cancer Center Central Germany (CCCG) Jena and Leipzig Germany; ^16^ Present address: Computational Medicine Center Thomas Jefferson University Philadelphia PA USA

**Keywords:** CD44 isoforms, colorectal cancer, HT‐29, metastasis

## Abstract

Hematogenous metastasis limits the survival of colorectal cancer (CRC) patients. Here, we illuminated the roles of CD44 isoforms in this process. Isoforms 3 and 4 were predominantly expressed in CRC patients. *CD44* isoform 4 indicated poor outcome and correlated with epithelial–mesenchymal transition (EMT) and decreased oxidative phosphorylation (OxPhos) in patients; opposite associations were found for isoform 3. Pan‐CD44 knockdown (kd) independently impaired primary tumor formation and abrogated distant metastasis in CRC xenografts. The xenograft tumors mainly expressed the clinically relevant CD44 isoforms 3 and 4. Both isoforms were enhanced in the paranecrotic, hypoxic tumor regions but were generally absent in lung metastases. Upon CD44 kd, tumor angiogenesis was increased in the paranecrotic areas, accompanied by reduced hypoxia‐inducible factor‐1α and CEACAM5 but increased E‐cadherin expression. Mitochondrial genes and proteins were induced upon pan‐CD44 kd, as were OxPhos genes. Hypoxia increased VEGF release from tumor spheres, particularly upon CD44 kd. Genes affected upon CD44 kd in xenografts specifically overlapped concordantly with genes correlating with *CD44* isoform 4 (but not isoform 3) in patients, validating the clinical relevance of the used model and highlighting the metastasis‐promoting role of CD44 isoform 4.

## Graphical abstract

Pan‐CD44 knockdown decreases spontaneous metastasis in human colorectal cancer xenograft models. Concurrent intratumoral gene expression alterations significantly correlate with genes differentially regulated among CD44 isoform 4 (but not isoform 3) high vs. low patients (TCGA). The corresponding gene sets and pathways include epithelial–mesenchymal transition, angiogenesis, and OxPhos. CD44 isoform 4 (but not isoform 3) correlates with poor patient outcomes.

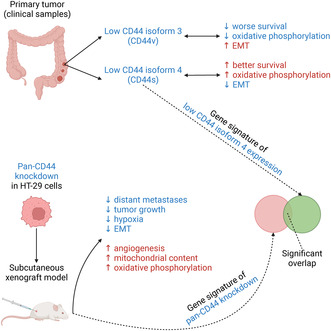

**Abbreviations**
CCLE: Cancer Cell Line Encyclopedia
CI: confidence interval
CM: conditioned media
CRC: colorectal cancer
CSC: cancer stem cell
DEG: differentially expressed gene
EC: endothelial cell
ECM: extracellular matrix
EMT: epithelial–mesenchymal transition
FDR: false discovery rate
FFPE: formalin‐fixed paraffin‐embedded
FPKM: Fragments Per Kilobase of transcript per Million mapped reads
GO: Gene Ontology
GSEA: Gene Set Enrichment Analysis
HA: hyaluronan
HIF: hypoxia‐inducible factor
HR: hazard ratio
HUVEC: human umbilical vein endothelial cells
kd: knockdown
Luc: luciferase
OAS: overall survival
OxPhos: oxidative phosphorylation
PFS: progression‐free survival
PT: primary tumor
RIN: RNA integrity number
s. c.: subcutaneous
SCID: severe combined immunodeficiency
TC: tumor cell
TCGA: The Cancer Genome Atlas
TMA: tissue microarrays
TMM: trimmed mean of M‐values

## 1 Introduction

The fate‐determining event for cancer patients is the development of distant metastases, as their presence is responsible for more than 90% of cancer‐related deaths [1]. Metastasis formation follows a complex cascade of individual steps that begin in the primary tumor (PT). Once the PT nodule reaches a certain size, it starts to emit angiogenic signals which in turn cause the growth of newly formed tumor blood vessels into the tumor [2]. Tumor vascularization is commonly aberrant so that solid tumors contain regions with transiently or permanently reduced oxygen and nutrient supply [3]. Hence, solid tumors often contain a necrotic center and an adjacent paranecrotic zone, which represents tumor cells (TCs) in a comparably hypoxic environment. Oxygen limitation is central not only in controlling glucose metabolism and survival of the TCs [4] but also in promoting epithelial–mesenchymal transition (EMT), which is orchestrated by hypoxia‐inducible transcription factors (HIFs) [5, 6]. EMT down‐regulates homophilic epithelial cell–cell contacts (like tight junctions and adherens junctions) and up‐regulates heterophilic tumor–endothelium cell adhesion molecules and/or their ligands, allowing extravasation and formation of distant metastases [7, 8, 9]. Furthermore, EMT leads to cytoskeletal rearrangements that improve cell motility and promotes anoikis resistance, which helps invading TCs survive their detachment from the basement membrane [10]. Moreover, EMT leads to the release of matrix‐degrading factors, further promoting invasion of the surrounding tissue. Thus, EMT is widely considered to be one key driver of metastasis formation [11, 12], particularly if cellular plasticity regarding the EMT status is provided that gives rise to hybrid E/M phenotypes [9, 13, 14, 15].

Once the TCs or small clusters of them break into tumor blood vessels, they have to survive in the bloodstream and attach to the endothelium of the target organ of the future metastasis [16]. The cancer cells achieve this by expressing glycoligands such as Sialyl Lewis A (SLe^A^), which are recognized by the endothelial (E/P‐) selectins that normally are essential in the adhesion and recruitment of leukocytes during inflammation [17, 18]. The selectin‐glycoligand interaction eventually results in an opening of the endothelial barrier, thus facilitating the leukocyte and TC endothelial transmigration [16]. Having entered the stroma of the target organ, TCs have to start to proliferate in order to grow to clinically detectable metastasis [19].

Cluster of differentiation 44 (CD44) is a transmembrane glycoprotein, also referred to as P‐glycoprotein 1. Due to its role in signal transduction, CD44 participates in normal cellular functions as well as tumor biological behaviors, including proliferation, differentiation, invasion, and motility [20, 21]. The expression of CD44 was shown to be aberrantly up‐regulated among several tumors, including colorectal cancer (CRC) [22, 23, 24, 25, 26, 27]. In the latter case, CD44 overexpression has been noticed as an early event occurring prior to the transformation of colorectal adenoma to carcinoma [28]. Importantly, CD44 is commonly accepted as a marker of cancer‐initiating cells (also known as cancer stem cells, CSC) [29, 30, 31] and of EMT [32, 33, 34]. Numerous studies demonstrate CD44 to be a potential therapeutic target among various cancers [21, 35].

From a functional point of view, CD44 has been described as a carrier of glycotopes that bind to E/L‐selectins, as observed in CD34+ hematopoietic stem cells, where sialofucosylated CD44 functions as a homing receptor and has been designated hematopoietic cell E/L‐selectin ligand [36]. While these glycoforms act as ligands to selectins, CD44 can also act as a glycoreceptor, namely by binding to hyaluronan (HA) [37], an extracellular matrix (ECM) glycosaminoglycan widely distributed within the connective tissues of mammals. In addition, CD44 has been shown to bind to collagens, osteopontin, matrix metalloproteases (reviewed in [38]), serglycin, fibronectin, and laminin (reviewed in [39]). All these functions would involve CD44 as a receptor within the metastatic cascade. In addition, the decoration of CD44 by selectin ligands would represent the ligand function of CD44, which would interact with the endothelial selectins [40, 41]. Hence, CD44 appears to be crucially involved in several steps of the metastatic cascade [38].

**Fig. 1:**
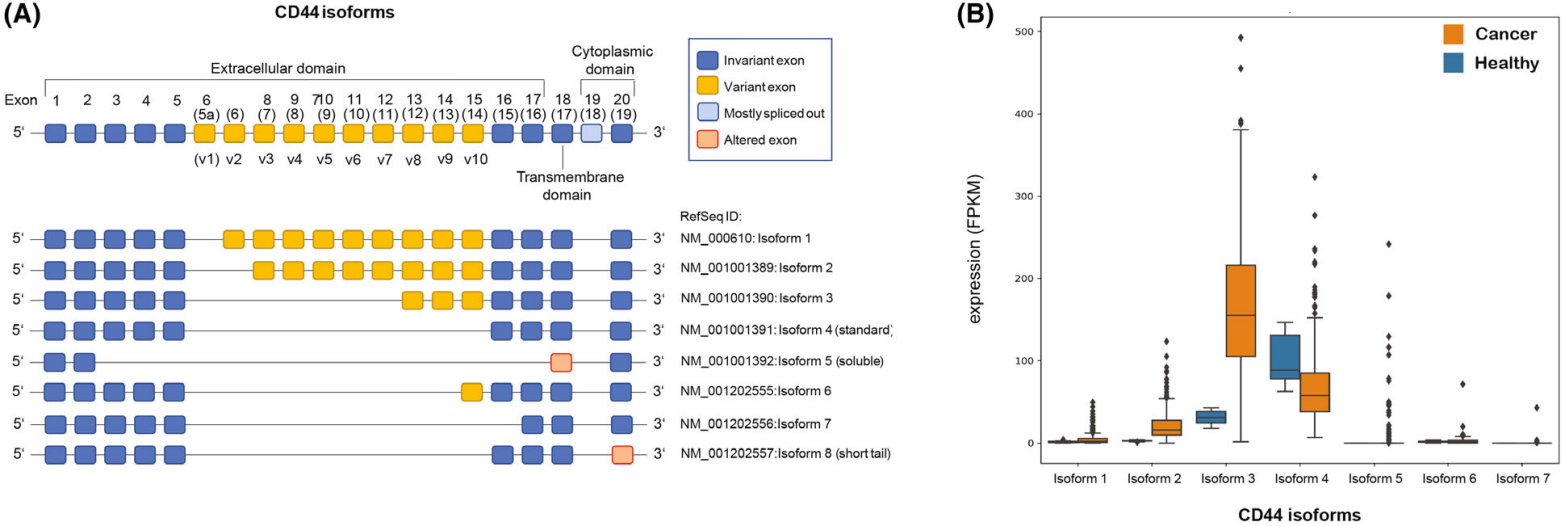
Expression of *CD44* isoforms in colon cancer PTs and tumor‐adjacent normal tissues. (A) Schematic illustration of known CD44 standard (blue) and variant (yellow) exons, their localization in the CD44 domains and their composition in the various CD44 isoforms. Exon 19 (light blue) is normally spliced out in humans. (B) Distribution of *CD44* mRNA isoforms' expression levels in PTs and adjacent normal tissues from TCGA‐COAD.

This marked multi‐functionality of CD44 is represented by multiple CD44 isoforms that have been discovered so far [21, 30, 31, 42, 43, 44]. *CD44* is encoded by 19 exons in humans, which undergo alternative splicing, giving rise to plentiful isoforms [44]. Among over 21 *in silico* predicted *CD44* isoforms, only eight have been experimentally confirmed [42] (see Fig. 1A for a summary of *CD44* isoforms). The CD44 standard isoform (CD44s, isoform 4) is constituted by the first five (invariant) exons 1–5 and the last four (invariant) exons. The latter ones are inconsistently numbered exons 15–17 and 19 or exons 16–18 and 20, depending on whether variant exon 1 (v1), which is not expressed in humans, is designated exon 5a or exon 6. Exon 18 (19) is mostly spliced out in humans. CD44s is ubiquitously expressed in most tissues. The inclusion of variant exons (CD44v2‐v10) results in larger isoforms which are expressed in only a few epithelial tissues, mainly in proliferating cells and in several cancers [20]. For instance, CD44 isoform 1 contains CD44v2‐v10, isoform 2 contains CD44v3‐v10 and isoform 3 contains CD44v8‐v10, each in addition to the invariant exons. Accumulating evidence supports the concept that CD44s and CD44v isoforms play different roles in cancer [21, 30, 31, 43]. For instance, CD44s (isoform 4) indicates a more mesenchymal TC phenotype while CD44v (e.g., isoform 3) a more epithelial phenotype, and isoform switches occur among the EMT [45, 46] and CSC spectrum [47], both of which crucially determine metastatic competence. However, the specific isoform expression patterns and functions lack extensive investigation for many types of tumors, including CRC, in which CD44 standard and variant isoforms containing exons v2, v3, v6, and v9 have been reported by others [27].

In the present study, we used a CRC xenograft model (HT‐29) representing the clinically most relevant CD44 isoforms (isoforms 3 and 4) to investigate the direct functional role of CD44 for spontaneous distant metastasis formation. Our multi‐omics‐based integrative analysis of xenograft and patient tissue suggests a strong pro‐metastatic role of CD44 isoform 4 in CRC. We further demonstrate that upon CD44 depletion, improved VEGF release and vascularization under hypoxic conditions are observed, accompanied by increased HSP60 (a marker for mitochondria) expression levels and oxidative phosphorylation (OxPhos) gene sets, as well as reduced EMT. Our findings support the notion that CD44 isoform 4 is an anti‐metastasis therapeutic target and give valuable insight into isoform specificity in CRC that is essential for unique cancer cell states and, thus, cancer phenotypes.

## 2 Materials and methods

### 2.1 The Cancer Genome Atlas (TCGA) and Cancer Cell Line Encyclopedia (CCLE) RNA sequencing data collection and processing

Bulk RNA sequencing tables of CRC PTs (*n* = 562) and adjacent normal tissues (*n* = 10) from TCGA‐COAD and TCGA‐READ cohorts were downloaded from Broad GDAC Firehose (https://gdac.broadinstitute.org/) in a format of splice‐variant read count matrices. Samples were divided into two groups of “left” and “right” CRC in the following way: biopsies taken from the rectum, splenic flexure, descending and sigmoid colon were marked as left‐sided cancer, while samples from the cecum, ascending colon, and hepatic flexure were marked as right‐sided cancer. In total, *n* = 163 samples were classified as left‐sided cancer, *n* = 127 were classified as right‐sided cancer, and a biopsy location was not available for *n* = 272 samples. Similar to the TCGA data, we downloaded splice‐variant‐level read count matrices for a total of *n* = 55 CRC cell lines from the CCLE [48].

The trimmed mean of M‐values (TMM) algorithm implemented in the edgeR v3.30.3 package [49] was used to normalize RNA‐seq count matrices into the TMM‐normalized Fragments Per Kilobase of transcript per Million mapped reads (FPKM) tables, and the log_2_ (FPKM +1) transformation was applied. Low‐expressed genes were detected using a standard edgeR background noise removal procedure.

### 2.2 Cell cultures and shRNA‐mediated CD44 knockdown

The human colon adenocarcinoma cell line HT‐29 (RRID:CVCL_0320) was purchased from the European Cell Culture Collection (Porton Down, Wiltshire, UK). Cells were grown in RPMI 1640 + l‐Glutamine (Gibco, Thermo Fisher Scientific, MA, USA) supplemented with 10% fetal calf serum (Gibco), 1% penicillin and streptomycin (Gibco) and cultured under standard cell culture conditions (37 °C, saturated humidity, 5% CO_2_). All cell lines have been authenticated in the past 3 years by STR profiling (DSMZ, Braunschweig, Germany). The HT‐29 cells were additionally tested for mycoplasma contamination.

CD44 knockdown (kd) in HT‐29 cells was achieved by a shRNA‐mediated approach: a 65 bp DNA oligomer containing a 19 bp anti‐*CD44* sequence (GGCGCAGATCGATTTGAAT) was inserted into the pLVX‐shRNA1 vector (Clontech, Takara Bio Europe, Saint‐Germain‐en‐Laye, France). The anti‐*CD44* sequence was checked for potential off‐target effects using NCBI BLAST (plus/plus and plus/minus strands), and no potential off‐target sequences with more than 14 identical bp (max. E value 25) were found. The same vector containing a sequence against firefly luciferase (Luc) was used to generate a transduced control cell line. Viral particles were produced as cell‐free supernatants by transient transfection of HEK‐293 T packaging cells as described [50]. In brief, lentiviral vectors based on pLVX‐shRNA1 were packaged using the second‐generation packaging plasmid psPAX2 (Addgene #12260, Addgene, Teddington, UK) and phCMV‐VSV‐G [51] expressing the envelope protein of vesicular stomatitis virus. The supernatant was harvested 24 h after transfection, 0.45 μm filtered and stored at −80 °C. Target cells were plated at 5 × 10^4^ cells in 0.5 mL medium in each well of a 24‐well plate. After the addition of viral particles containing supernatant to the cells, the medium was replaced the next day and puromycin was added the second day after transduction at a concentration of 1 μg·mL^−1^. The puromycin selection was carried out for at least 1 week.

The efficiency of CD44 kd was confirmed by flow cytometry. The same construct has been tested and successfully employed in two other cell lines and corresponding xenograft models, specifically HOS osteosarcoma and MeWo melanoma cells/xenografts [41].

### 2.3 Flow cytometry

For the detection of pan‐CD44 (Diaclone, clone B‐F24, #852.601.010), CD44v3 (R&D systems, clone 3G5, #FAB5088A), CD44v4 (Novus, clone CD44v4/1219, #NBP2‐54581AF488), CD44v4/5 (R&D systems, clone 3D2, #FAB5399P), CD44v5 (Novus, clone VFF‐8, #NB100‐65533G), CD44v6 (Invitrogen, clone VFF‐7, #AHS4468), CD44v7/8 (Novus, clone VFF‐17, #NB100‐65535G) and CD44v9 (Novus, clone CD44v9/1459, #NBP2‐54571AF488), subconfluently grown TCs were detached using enzyme‐free Cell Dissociation Buffer (Gibco, #13150016). The expression of SLe^A^ (Novus, clone 121SLE, #NBP2‐54349G) or static binding of recombinant human E‐selectin (rhE‐selectin/human IgG Fc chimera, R&D Systems, #724‐ES) was determined after detachment with trypsin. 0.5–1 × 10^6^ cells were stained with aforementioned antibodies or fluorescence‐labeled E‐selectin chimera (labeled as described before, [52]) in a final concentration of 1 μg·mL^−1^ in 100 μL FACS buffer (PBS^−CaCl2/−MgCl2^ + 1% BSA + 0.05% NaN_3_; for E‐selectin binding PBS^+CaCl2/+MgCl2^ + 1% BSA + 0.05% NaN_3_) for 15 min on ice and measured by flow cytometry (MACSQuant Analyzer 10 or Sysmex CyFlow Cube 8). Propidium iodide was used for live/dead distinction, and data analysis was carried out with the software flowlogic™ V.8 (inivai, Mentone VIC, Australia) for measurements with the macsquant Analyzer and fcs express 4 (*De Novo* Software, Los Angeles, CA) for measurements with CyFlow Cube 8.

### 2.4 Animal experiments

The methodology for carrying out the animal experiments was consistent with the UKCCR guidelines for the welfare of animals in experimental neoplasia [53]. The experiment was supervised by the institutional animal welfare officer and was approved by the local licensing authority (Behörde für Soziales, Familie, Gesundheit und Verbraucherschutz; Amt für Gesundheit und Verbraucherschutz, Hamburg, Germany) under the project no. G10/100.

All animals used were pathogen‐free Balb/c severe combined immunodeficient (SCID) mice (nomenclature: CB17/Icr‐Prkdc^scid^/IcrIcoCrl; source: breeding facility of the Forschungstierhaltung at UKE, Hamburg, Germany) from both sexes aged 9–14 weeks with a weight of 25–30 g at the beginning of the experiment. The mice were housed in filter‐top cages, provided food and water *ad libitum*, and their condition was monitored daily. Each animal was inoculated subcutaneously (s. c.) above the right scapula with 1 × 10^6^ HT‐29 Luc or HT‐29 CD44 kd cells in a medium without supplements. The mice were sex‐matched across the HT‐29 Luc and HT‐29 CD44 kd groups.

Apart from the visible s. c. tumors, the general condition of the animals was evaluated by a standardized in‐house scoring system based on movement/behavior, weight development, food and water intake and fur condition. The mice were euthanized when the tumors ulcerated, reached a size of ~ 1.5 cm^3^ or when the mice showed a change of their initial body weight of > 10%. For necropsy, mice were anesthetized by intraperitoneal injection of a weight‐adapted dose (10 μL·g^−1^ body weight) of a mixture of 1.2 mL Ketamin (Gräub AG, Bern, Switzerland), 0.8 mL Rompun (Bayer AG, Leverkusen, Germany) in 8 mL saline. Blood samples were then collected by cardiocentesis into EDTA tubes for DNA extraction and subsequent quantitative real‐time PCR for human‐specific *Alu* sequences (*ALU*‐PCR, see below), and mice were sacrificed by cervical dislocation. Livers and lungs were excised at necropsy for subsequent histology (largest liver lobe, right lung) or DNA extraction (remaining liver lobes, left lung). The femora and tibiae of the mice were dissected, opened transversely at the metaphyses and flushed with 500 μL 0.9% NaCl solution to harvest the bone marrow for DNA extraction. Finally, s. c. PTs were dissected and cut into four pieces for histology and proteomics (two separate samples fixed in 4% formalin for 24 h), as well as RNA sequencing and kinomic profiling (two separate samples stored in liquid nitrogen).

The spontaneous metastatic cell loads in the lung, liver, and bone marrow samples were quantified by *ALU*‐PCR as described [54, 55, 56]. For histological quantification of lung metastases, formalin‐fixed, paraffin‐embedded (FFPE) lung samples were serially sectioned, ten representative sections from the middle of the lungs stained HE, and the number of metastases counted in these ten sections per mouse using a light microscope as described [57].

During the project, we later aimed to analyze initial vs. established spontaneous lung metastases from the s. c. HT‐29 xenograft model by immunohistochemistry. For this purpose, the described animal experiment was repeated in a modified way: 1 × 10^6^ HT‐29‐Luc2/RGB cells were s. c. injected into SCID mice, and PTs were surgically resected at a size of ~ 1 cm^3^ as described [58]. After a post‐surgical observation period until recurrent tumors reached again ~ 1 cm^3^, mice were examined for lung metastases by bioluminescence imaging (IVIS 200, Perkin Elmer, Waltham, MA, USA), sacrificed, lungs excised and processed for subsequent immunohistochemistry as described above. These experiments were approved by the local authorities (Behörde für Justiz und Verbraucherschutz der Freien und Hansestadt Hamburg, Lebensmittelsicherheit und Veterinärwesen, Hamburg, Germany) with the project no. G16/55.

### 2.5 Immunohistochemistry and quantification

All immunohistochemical staining procedures were carried out on dewaxed paraffin sections. The staining protocols relevant to this study are briefly summarized in Table S1. Staining results were quantified only when differences were apparent in the initial analysis of the total tissue area by light microscopy. In such a case, at least four tumors per group were considered, stained sections digitized (Zeiss AxioScan.Z1, Zeiss, Jena, Germany), and images taken in at least five viewing fields each (depending on the size of the tumor) from both the paranecrotic region and the tumor margin. After setting a staining‐specific threshold for red color, the percentage of stained pixels per image was automatically measured using imagej version 1.53c [59].

An identical procedure was followed for staining E‐cadherin and CEACAM5 in spontaneous lung metastases. Stained sections were digitized, individual lung metastases marked, and their size [μm^2^] was determined by using the freehand selection tool in imagej. Afterward, the percentage of the stained area was measured as described above, but specifically only within the annotated metastasis area. The mCD31 staining was evaluated by manually counting the number of mCD31+ microvessels per field of vision.

To generate positive control samples for hypoxia‐inducible factor (HIF)‐1α and HIF‐2α immunohistochemistry, routine cultured HT‐29 cells were transferred to hypoxic conditions (1% O_2_) for 24 h in a Steri‐Cycle i160 CO_2_ incubator (Thermo Fisher) and afterward embedded in agar/paraffin.

### 2.6 RNA sequencing

Approximately 50 mg of fresh‐frozen xenograft PT tissue was crushed in liquid nitrogen in the presence of QIAzol Lysis Reagent (Qiagen, Hilden, Germany). The total RNA was isolated using miRNeasy Mini Kit (Qiagen, Hilden, Germany) according to the manufacturer's instructions. All RNA samples were treated with DNase I during the isolation procedure. The RNA yield was determined by UV absorbance using a NanoDrop 1000 spectrophotometer (Peqlab, Erlangen, Germany). The RNA quality was assessed by analyzing the ribosomal RNA integrity number (RIN) on an Agilent 2100 Bioanalyzer using the RNA 6000 Nano kit (Agilent Technologies, Palo Alto, CA, USA). The RIN values of the isolated RNA samples ranged from 6.7 to 9.6. Libraries for mRNA sequencing were prepared from total RNA samples using the Illumina Stranded mRNA Library Prep Kit (Illumina, San Diego, CA, USA) according to the manufacturer's instructions. Each sample was sequenced on the Illumina NextSeq 550 to generate paired‐end reads.

The quality of RNA‐seq FASTQ files was assessed with FastQC v0.11.9 (Babraham Bioinformatics, Cambridge, UK). Adapter sequences were removed with the use of cutadapt v2.10 [60]. Adapter‐free RNA‐seq reads were mapped to the reference human genome (GENCODE GRCh38.p13) with STAR v2.7.5b [61]. GENCODE genome annotation (release 34) was used to generate the gene‐level read counts matrices. To quantify the expression levels of *CD44* splice isoforms, we used essentially the same pipeline except for the read mapping step, which was done with the use of Salmon v1.2.1 [62]. Normalization of RNA‐seq data was performed using edgeR similar to the TCGA and CCLE data.

### 2.7 RT‐qPCR

RNA was reverse transcribed to cDNA using 500 ng of total RNA as a starting material and SuperScript VILO cDNA Synthesis Kit (Invitrogen, Carlsbad, USA) according to the manufacturer's recommendations. Quantitative PCR analysis was carried out using the SYBR Green 5x qPCRmix‐HS SYBR reaction mix (Evrogen, Moscow, Russia) as described in [63]. Primer pairs were designed and characterized as described in [64]. Primer sequences are presented in Table S2. PCR efficiencies of all primer sets were higher than 1.9 and lower than 2.11. All RNA samples were analyzed in triplicate and averaged. Target gene expressions were normalized to the reference genes *PTMA*, *SF3A1*, *HPRT1*, and *MRPL19*, and data were processed based on the ∆∆*C*_t_ method [65]. Reference gene selection and validation were performed using the approach described in [66].

### 2.8 Proteomic analysis

Formalin‐fixed paraffin‐embedded tissue sections (10 μm) from each xenograft tumor block were inserted into a 1.5 mL microcentrifuge tube and washed sequentially with dewaxing solvents with mild agitation. Between each step every sample was centrifuged for 5 min to remove the supernatant: xylene (1 × 5 min; 100 μL), 100% ethanol (1 × 2 min; 100 μL), 95% ethanol (1 × 2 min; 100 μL), 70% ethanol (1 × 2 min; 100 μL), 50% (1 × 2 min; 100 μL), water (1 × 2 min; 100 μL) and after the last centrifugation, the pellet was dried under vacuum. For antigen retrieval, dewaxed FFPE sections were incubated in 0.1 m Tris–HCl, pH 8, 0.1 m DTT (100 μL), incubated for 2 h, 60 °C, with agitation at 600 rpm and then further sonicated for 3 min. Following the sonication, 10 mm of iodoacetamide was added to the microcentrifuge for 30 min at room temperature in the dark. After the incubation, 2 μg of trypsin was added and incubated overnight, 37 °C with gentle shaking. To extract the digested peptides, samples were centrifuged (5 min, 11 337 ***g***), and the supernatant was transferred into a new tube. FFPE scrolls were washed again with 100 μL (20% ACN + 0.1% TFA), centrifuged (5 min), and the supernatants were combined and dried under vacuum.

A portion of each sample was concatenated for further offline peptide fractionation. Peptides were resuspended in 350 μL of 0.1% TFA and fractionated using a Pierce™ High pH Reversed‐Phase Peptide Fractionation Kit (Sigma, Australia) following the manufacturer's instructions. Briefly, the peptides were loaded to the pre‐conditioned supplied spin columns and washed (3000 ***g***, 2 min) once using water. Increasing concentrations of acetonitrile (ACN) (5%, 7.5%, 10%, 12.5%, 15%, 17.5%, 20%, and 50%) in 0.1% triethylamine (TEA) buffer were used to elute (3000 ***g***, 2 min) the bound peptides into eight distinct fractions. The resulting fractions were dried under a vacuum.

Peptides were loaded onto a pepmap pre‐column with a 75 μm inner diameter. The column temperature was maintained at 45 °C with a column oven. A Dionex UltiMate 3000 RSLCnano HPLC system (Thermo Fisher Scientific, USA) was interfaced with an Orbitrap Fusion mass spectrometer (Thermo Fisher Scientific) using a Nanospray Flex ion source (Thermo Fisher Scientific, USA). The peptides were separated over a monocap C18 nano‐flow column (0.1 × 150mm; GL Sciences, CA, USA) with a binary buffer system of 0.1% (vol/vol) formic acid (buffer A) and 80% (vol/vol) acetonitrile/0.1% (vol/vol) formic acid (buffer B), and eluted at a flow rate of 300 nL/min over a 130 min LC run. An initial BoxCar‐DDA library was generated using the offline fractionated peptides. The MS1 scans were recorded from 350 to 1650 m/z recorded at 120 000 resolution with an AGC target of 250% and a maximum injection time of 246 ms. The Boxcar segments with their associated variable widths were adapted from Sinitcyn et al. [67]. The BoxCar scans comprised 24 segments of variable width, with three BoxCar scans (multiplexed targeted SIM scan) isolating eight segments per scan. The MS2 scan segments were identical to the MS1 scans. Precursor ions were isolated at 1.6 isolation width and accumulated for a maximum of 22 ms, and the normalized AGC target was set to 100%. Fragmentation was induced with stepped HCD of collision energy of 25%, 35% and 50%. The MS2 scans were measured at a resolution of 15 000 at m/z 200. Only precursors with charge states 2–10 were selected; unassigned charge states were excluded. Dynamic exclusion of targeted precursors was set for 60 s. Similarly, a BoxCar DIA analysis was carried out for each sample without any fractionation. The LC and column setup were identical to the DDA analysis. The BoxCar MS1 and MS2 segments used were also identical; a 1‐m/z overlap was retained between boxes in adjacent scans. BoxCar MS1 scans were also recorded at a resolution of 120 000, with the normalized AGC target set at 200% per segment, with a maximum injection time of 246 ms. MS2 scans of 24 segments were recorded at a resolution of 30 000 and an AGC target of 2000%, with a maximum injection time of 60 ms. Fragmentation was induced with stepped HCD of collision energy of 22%, 27%, and 32%. All raw datasets generated for this current study have been deposited to the ProteomeXchange Consortium with the dataset identifier.

Raw MS data were processed using Spectronaut version 15.1.210713.50606 (Biognosys AG) [68] with the default settings, using a spectral library generated from the offline peptide fractionated BoxCar DDA raw files. A maximum of 2 missed trypsin cleavages, cysteine carbamidomethylation as fixed modification, and methionine oxidation as variable modification was set. The acquisition data were searched against a Human (UP000005640) and Mouse (UP000000589) database. Data were filtered out at a false discovery rate (FDR) of 1% or at *q*‐value <0.01 at the protein level.

### 2.9 Differential expression and enrichment analyses

DESeq2 v1.28.1 [69] was used to carry out the differential expression analysis for RNA‐seq data. For the proteomics data, we used Student's *t*‐test. The obtained *P*‐values were adjusted by the Benjamini‐Hochberg procedure. Significantly differentiated genes were identified by setting a 0.05 threshold on FDR and a minimum absolute fold change of 1.5.

Enrichment analysis for transcriptome and proteome data upon CD44 kd was performed using DAVID 2021 [70] with Gene Ontology (GO) pathway set, and Gene Set Enrichment Analysis (GSEA) [71] with hallmarks pathway set. We executed GSEA using the gseapy python package (https://gseapy.readthedocs.io). Significantly enriched terms were identified by setting a 0.05 threshold on FDR.

The described differential expression analysis was also used to rule out possible off‐target effects of the used shRNA. Firstly, RNA22 v2.0 [72] was used to predict the siRNA's target genes based on its sequence. Then, the hypergeometric test was used to assess whether the predicted targets were overrepresented among differentially expressed mRNAs and proteins. As a result, no statistically significant enrichment was found: *P* = 0.74 for the transcriptome and *P* = 0.6 for the proteome. In addition, no significant enrichments were found when the sets of down‐regulated and up‐regulated mRNAs and proteins were considered separately (*P* > 0.19).

### 2.10 HA staining

To visualize the presence and distribution of HA in the xenograft PT tissues, deparaffinized sections were pretreated with antigen retrieval solution (Dako #S1699) at 60 °C in a water bath overnight, followed by a blocking step with 1% BSA in Tris‐buffered saline for 30 min at room temperature (RT). Sections were then incubated for 1 h at RT with biotinylated HA‐binding protein (HABP, Calbiochem #385911), diluted 1 : 75 in Dako antibody diluent. Binding was detected using the Vectastain ABC AP kit (Vector Labs) and Liquid Permanent Red solution (Dako) according to the manufacturers' instructions. Nuclei were counterstained with Mayer's hemalum solution for 3–5 s.

The Masson‐Goldner trichrome staining was performed according to a routine protocol.

### 2.11 Kinome profiling

Analysis of kinase activity in xenograft tumor tissue was performed as described previously [73] using a PamStation®12 (located at the UCCH Kinomics Core Facility, UKE, Hamburg, Germany) according to the manufacturer's instructions (PamGene International, 's‐Hertogenbosch, The Netherlands). In brief, for profiling protein tyrosine kinases (PTK), PTK‐PamChip® arrays were used. Each array contains 140 individual peptide sequences derived from substrates for tyrosine kinases. Whole cell lysates were made using M‐PER Mammalian Extraction Buffer containing Halt Phosphatase Inhibitor and EDTA‐free Halt Protease Inhibitor Cocktail (Pierce, Waltham, MA, USA; Cat # 78440). Per array, 5 μg of protein and 400 μM ATP were applied. Sequence‐specific peptide tyrosine phosphorylation was detected by the fluorescein‐labeled antibody PY20 (Exalpha, Maynard, MA, USA; Cat # X1017S) and a CCD camera using the evolve software (PamGene International). After quality control, the final signal intensities were log_2_‐transformed and were used for further data analysis using the bionavigator software version 5.1, including the upstream kinase analysis app (PamGene International).

### 2.12 Tissue microarrays

The tissue microarrays (TMA) used in this study contained retrospectively collected samples (1995 to 2008) from CRC patients (*n* = 58) where PT tissue as well as adjacent normal tissue and tissue of resected liver metastases were available from the University Medical Center, Göttingen, Germany. The study methodologies conformed to the standards set by the Declaration of Helsinki. The experiments were undertaken with the understanding and written consent of each subject. The study methodologies were approved by the Ethics Committee of the University Medical Center, Göttingen, Germany. TMAs were generated with a core needle diameter of 1 mm and in accordance with the local ethics committee approval (application number 21/3/11). Deparaffinized freshly cut sections of TMAs were rehydrated and treated with the staining protocols for HIF‐1α, HSP60, or CD44v9, as summarized in Table S1. Expression intensity was examined in a semiquantitative manner as described before (score 0: no staining, score 1: weak, score 2: moderate, and score 3: strong staining) [74]. In the case of HSP60, there was no negative staining (scores ranging from 1 to 3). All TMA spots that contained TCs were included in the analysis. If more than one TMA spot from an individual patient contained TCs, the mean value of the staining score was included for this patient in the analysis.

### 2.13 Survival analysis

Python Lifelines implementation of the Cox regression model, log‐rank test, and Kaplan–Meier estimation were used for the survival analysis [75]. Kaplan–Meier survival curves with a log‐rank test were plotted for *CD44* isoform 3 or isoform 4 low and high expression groups formed in the following way: each quantile level (from 0.1 to 0.9 with a 0.01 step) of isoform expression was set as a dividing threshold, and the log‐rank test statistic was calculated. Further, the expression level corresponding to the highest obtained test statistic was selected as the optimal dividing threshold (further referred to as a cutoff point). As a result, samples with isoform expression higher than the cutoff point were marked as a high expression group, while the rest of the samples were marked as a low expression group. The statistical significance of a found difference between these groups was evaluated via the null hypothesis that the data has an equal overall survival (OAS) rate between groups formed by any expression‐dividing threshold. To test the hypothesis, the permutation test was implemented in the following way: first, the log‐rank test statistic was calculated for the previously selected optimal cutoff point. Second, survival data (event and time to event) of all patients was randomly mixed, and the optimal cutoff point with the related log‐rank test statistic was calculated. The latter procedure was performed multiple times (*N* = 10 000), and the fraction of obtained statistic values greater than the initial value was assessed. A low fraction value (*P* < 0.05) implies a rejection of the null hypothesis, indicating a low probability of the random effect for the selected optimal cutoff point. Hazard ratio (HR) scores with their 95% confidence intervals (CI) were calculated using an univariate Cox regression model fitted with log_2_‐transformed expression values, reflecting a change in survival probability associated with a 2‐fold increase in expression level.

Considering that *CD44* isoforms 3 and 4 share similar structure as well as have similar expression levels in CRC tissues, our next goal was to find a universal expression threshold level corresponding to the largest divergence of Kaplan–Meier curves for low and high expression groups for both isoforms together. Based on the previously described pipeline, we maximized the average log‐rank test statistic for these groups and found a large set of expression levels that produced a statistically significant difference in OAS rates. In particular, the log_2_‐transformed expression level of 6.5 was obtained as the optimal dividing threshold level for *CD44* isoform 3 and isoform 4 (avg. *P*‐value < 0.01). Further, samples with expression levels of isoforms 4 or 3 lower than the aforementioned threshold are referred to as *CD44* isoform 4 low or *CD44* isoform 3 low, while the rest of the patients are referred to as *CD44* isoform 4 high or *CD44* isoform 3 high.

### 2.14 Comparison of sets of differentially expressed genes (DEGs)

To compare two sets of DEGs, the following Monte‐Carlo procedure was applied. First, two sets of randomly selected genes with the corresponding cardinalities were generated from all protein‐coding genes. Second, each gene was uniformly assigned a sign +1 or − 1 to consider the direction of change. Then, the cardinality of the intersection of these two sets was compared to the cardinality of the intersection of the initial two sets of DEGs. This procedure was performed multiple (*N* = 10 000) times, and the resulting *P*‐value was calculated as the percentage of intersections with cardinalities greater than the initial one.

### 2.15 3D sphere formation assay in normoxia and hypoxia

For subsequent flow cytometric analyses, 3D spheres of HT‐29 Luc and CD44 kd cells were generated by cultivation on Poly‐2‐hydroxyethyl methacrylate (PolyHEMA)‐coated culture flasks. PolyHEMA (Sigma, St.Louis, MO, USA) was dissolved to 12 mg·mL^−1^ in 95% absolute ethanol. Culture flasks were covered with PolyHEMA solution and incubated at 37 °C in a dry atmosphere until the liquid evaporated. Afterward, 1 × 10^6^ TCs per flask were seeded in a 10 mL cell culture medium and cultured for 6 days. One part of the established tumor spheres was cultivated under hypoxic conditions for the last 24 h of cultivation. Afterward, tumor spheres were harvested, enzymatically and mechanically dissolved, and filtered to obtain a single‐cell solution, and this solution was subjected to staining for flow cytometry.

For assessing the colony forming capacity, VEGF release and indirect effects on EC tube formation, a 1 : 2 mixture of matrigel and cell suspension (in medium with 0.2 mg·mL^−1^ HA) of 1200 TC·mL^−1^ was prepared (final concentration of 600 TC·mL^−1^ in medium/matrigel containing 0.1 mg·mL^−1^ HA) and given to 96‐well plates (50 μL per well) to enable colony formation. The wells were cultivated under normoxic or hypoxic conditions for the last 36 h of 9 days incubation time in total. On day nine, formed spheres were photographed using a light microscope, and their average size per well [colony area in (μm^2^)] was quantified using imagej. In addition, the corresponding cell culture supernatants (conditioned media, CM) of each well were harvested and analyzed using a VEGF ELISA kit (R&D systems, #DY293B) according to the manufacturer's instructions. Photometric measurements were carried out using an MAX 002 plate reader (Dynex Technologies). The detected VEGF concentrations were then normalized to the sums of all colonies visible in the respective wells. Additional CM samples were used for endothelial cell (EC) tube formation assays.

### 2.16 EC tube formation assay

The effect of soluble factors secreted by Luc vs. CD44 kd 3D tumor spheres under normoxic vs. hypoxic conditions on the angiogenic behavior of EC (human umbilical vein ECs, HUVEC; PromoCell) was measured by a tube formation assay. For this purpose, 80 μL of Matrigel® Growth Factor Reduced (Corning) was applied to a 96‐well plate, and 1.5 × 10^4^ HUVEC were added in 100 μL of CM from HT‐29 Luc or CD44 kd tumor spheres grown in Matrigel/HA (see above) and cultivated for 4 h in the presence of 10% FCS. Images were acquired using the Zeiss ApoTome microscope and a 10x A‐Plan Ph1 objective (NA: 0.25, WD (mm): 4.5). Tube formation was assessed by measuring the total tube area per well [μm^2^] with Fiji imagej software.

### 2.17 TC proliferation, migration, and invasion assays

To analyze TC proliferation under subconfluent two‐dimensional conditions, 5 × 10^4^ TCs were seeded into T25‐flasks, and the resulting cell number was manually counted in a Neubauer counting chamber on d1‐4. The experiment was run in biological triplicates.

For studying the migratory and invasive behavior of TCs, Corning^®^ Fluroblok™ and Corning^®^ Biocoat™ FluroBlok™ transwell assays (8 μm pore size), respectively, were used according to the manufacturer's instructions (VWR, Darmstadt, Germany). Migrated/invaded cells were quantified based on the relative fluorescence units (RFU) measured in a Tecan Genios microplate reader (Tecan, Männedorf, Switzerland).

### 2.18 Laminar flow adhesion assay

Laminar flow adhesion assays with HT‐29 Luc vs. CD44 kd cells on HUVEC monolayers were performed as previously described [41, 76].

### 2.19 Statistical analysis

Correlation analysis was performed using python scipy [77] implementation of Spearman's rank correlation with the additional Benjamini‐Hochberg *P*‐value adjustment. Absolute correlation scores greater than 0.3 and FDR lower than 0.05 were considered as significant. The independence of groups of ordinal values was evaluated with the use of Pearson's chi‐square test. Survival analyses of patient (TMA) data were carried out using the Log‐rank (Mantel‐Cox) test.

Animal experiment data were analyzed using the Wilcoxon rank sum test with continuity correction (tumor weight, lung metastasis number by histology) or the Wilcoxon rank sum exact test (all *ALU*‐PCR data). The association between tumor weight, CD44 kd and metastasis numbers was calculated using a multiple linear regression model, including covariates for tumor weight and CD44 kd status. The analyses were performed with r version 4.2.1 [78].

## 3 Results

### 3.1 *CD44* isoform expression patterns in CRC patient tissues

A schematic illustration for the various *CD44* isoforms is presented in Fig. 1A. By analyzing the *CD44* isoform mRNA expression patterns in TCGA cohorts, we revealed a 7‐fold increase of *CD44* isoform 3 in cancerous as compared to healthy control tissue of the colon (Fig. 1B). Isoform 4 also showed a relatively high median expression in tumor tissue, but its expression was almost 2‐fold decreased in comparison to normal tissue (Fig. 1B). As mentioned above, isoform 3 contains variant exons CD44v8–v10 in addition to the invariant standard exons. Isoform 4 (*CD44s*) contains invariant exons only (Fig. 1A). Isoforms 1, 2, and 5–7 were detectable at low levels or nearly not detectable. Isoform 8 is not available in TCGA. Concordantly with the cancerous up‐regulation of *CD44* isoform 3 (the most highly expressed isoform), total *CD44* levels were also increased in tumor samples (Fig. S1A). There was no significant difference in total *CD44*, *CD44* isoform 3 or *CD44* isoform 4 expression levels between CRC stage I‐IV (Fig. S1A–C). Isoforms containing variant exons v8‐v10 (isoforms 1–3) strongly correlated positively with each other, while isoform 3 had a weak negative correlation with isoform 4 (*r* = −0.27, Fig. S1D).

### 3.2 CD44 isoforms 3 and 4 are oppositely associated with the prognosis of CRC patients

**Fig. 2:**
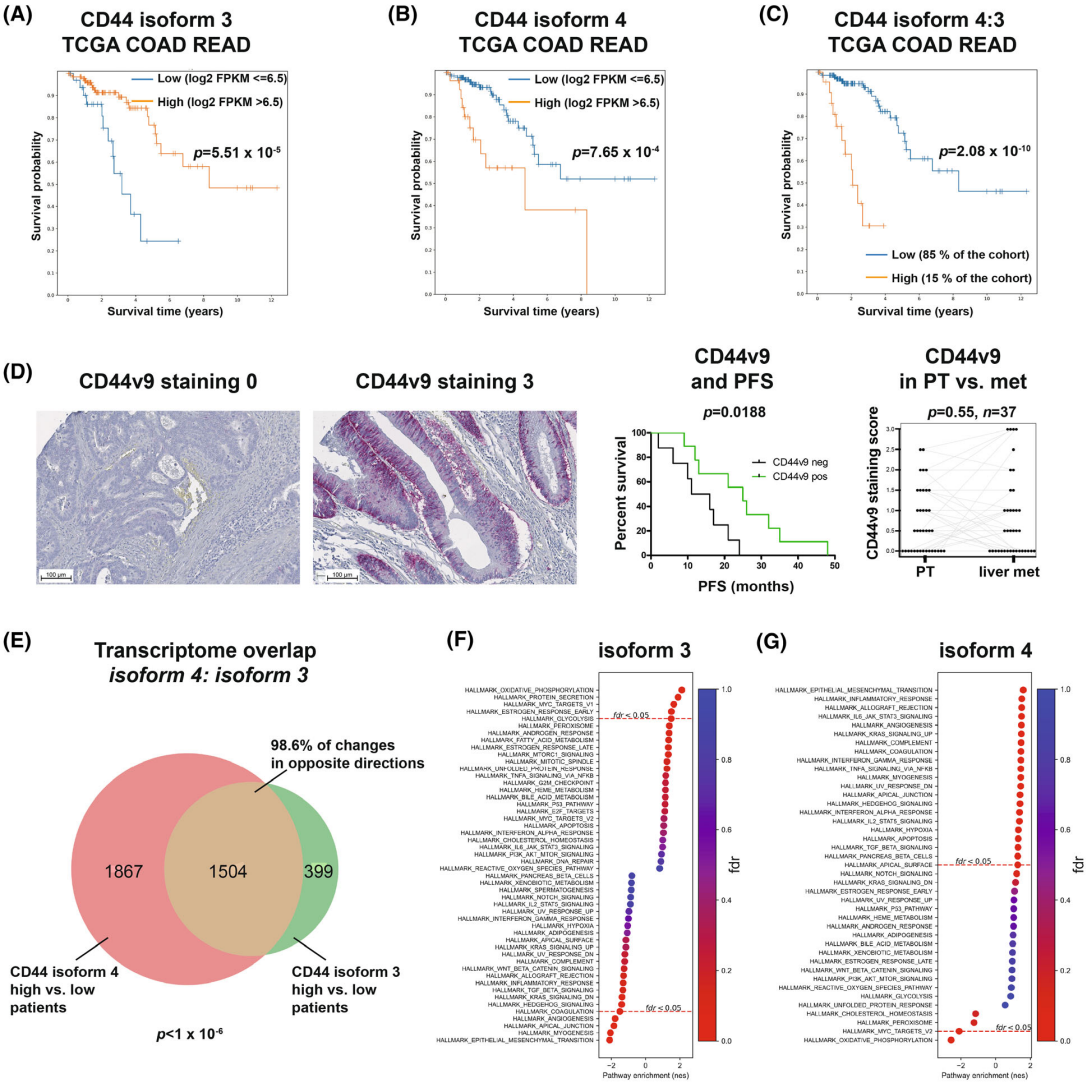
Prognostic role of CD44 isoforms 3 and 4 and their correlation with other genes' expression levels in colon cancer. (A–C) OAS of left‐sided colon cancer patients stratified for high vs. low CD44 isoform 3 (A) or isoform 4 expression (B) or isoform 4:3 ratio (C). (D) PFS (time from PT surgery to occurrence of metachronous liver metastases) of CD44v9‐negative vs. ‐positive CRC patients and CD44v9 levels in matched pairs of CRC PT and liver metastasis (Met) samples. Two slides on the left show representative examples of CD44v9 negative (score 0) and positive tumors (score 3), scale = 100 μm. (E) Venn diagram showing transcriptional overlap of the DEGs between CD44 isoform 4 high vs. low patients and CD44 isoform 3 high vs. low patients (TCGA COAD and READ datasets). Note that almost all overlapping genes change their expression in opposite directions (98.6%). (F, G) GSEA of genes differentially expressed among left‐sided colon cancer patients with high vs. low CD44 isoform 3 or isoform 4 expression as indicated. Note that EMT and angiogenesis gene sets are significantly down‐regulated in the CD44 isoform 3 high group, while the OxPhos gene set is up‐regulated (F). In contrast, EMT is up‐regulated, and OxPhos is down‐regulated in the CD44 isoform 4 high group (G). The following statistical tests were used: log‐rank test (A, B, C, “CD44v9 and PFS” panel in D), paired Student's *t*‐test (“CD44v9 in PT vs. met” panel in D), hypergeometric test (E).

We then analyzed the distribution of *CD44* isoforms in left and right‐sided CRC tissues (TCGA) because accumulating evidence in the literature suggests that biological differences exist between both sides [79, 80]. In our study, there was no statistically significant difference in isoform expression between both sides (Fig. S2). Nevertheless, Cox survival regression analysis for the left‐sided CRC samples revealed a better OAS for patients with higher expression of isoform 3 (HR = 0.2, CI = 0.1–0.5, *P* = 5.51e‐5; Fig. 2A). In contrast, patients with higher expression of isoform 4 had a worse OAS outcome (HR = 3.3, CI = 1.6–6.9, *P* = 7.65e‐4; Fig. 2B). Since isoform 4 and 3 were negatively correlated and contributed in opposite ways to OAS, we found that a higher isoform 4:3 ratio resulted in an even more striking association with poor prognosis (HR = 9.8, CI = 4.2–23.1; *P* = 2.1e‐10, Fig. 2C). Notably, there was no statistically significant link between expression of *CD44* isoforms and OAS for the right‐sided CRC samples (data not shown). Furthermore, expression of total *CD44* mRNA was not prognostic in both parts of the colon, suggesting the particular importance of *CD44* splicing (data not shown).

To validate these results on the protein level, we analyzed whether the CD44v9 (includes isoform 3) level in CRC patient PTs plays a role in progression‐free survival (PFS, time from PT surgery to the occurrence of metachronous liver metastases). Concordantly with TCGA mRNA data, PFS was shorter in patients with negative CD44v9 protein expression (median: 13.5 months) than in patients with positive CD44v9 protein expression (median: 25.0 months, *P* = 0.0188, Log‐rank (Mantel‐Cox) test, Fig. 2D). CD44v9 levels did not change in liver metastases compared to matched PTs (*P* = 0.55, paired *t*‐test, Fig. 2D).

To discover possible mechanisms responsible for the divergent prognostic effects of the isoforms, we assessed differences in transcriptomic landscapes of low and high‐expression patient groups using differential expression and gene enrichment analysis. In total, we identified 1903 DEG for the *CD44* isoform 3 high vs. low groups (Table S3) and 3371 DEGs for the *CD44* isoform 4 high vs. low groups (Table S4). A large portion of genes (*n* = 1504) changed in both cases, 1483 of which changed in opposite directions (Fig. 2E). GSEA of low and high expression groups revealed a number of significantly enriched pathways (*n* = 23 for isoform 4 and *n* = 10 for isoform 3). Notably, according to the direction of change of overlapping genes, *n* = 6 pathways were enriched in opposite directions in both cases. Among them, EMT was significantly down‐regulated in the *CD44* isoform 3 high group (Fig. 2F) and significantly up‐regulated in the *CD44* isoform 4 high group (Fig. 2G). Similarly, we found that the OxPhos pathway was regulated in opposite directions: up‐regulated in the *CD44* isoform 3 high group (Fig. 2F) and down‐regulated in the *CD44* isoform 4 high group (Fig. 2G).

### 3.3 CD44 regulates tumor growth and spontaneous distant metastasis *in vivo*

**Fig. 3:**
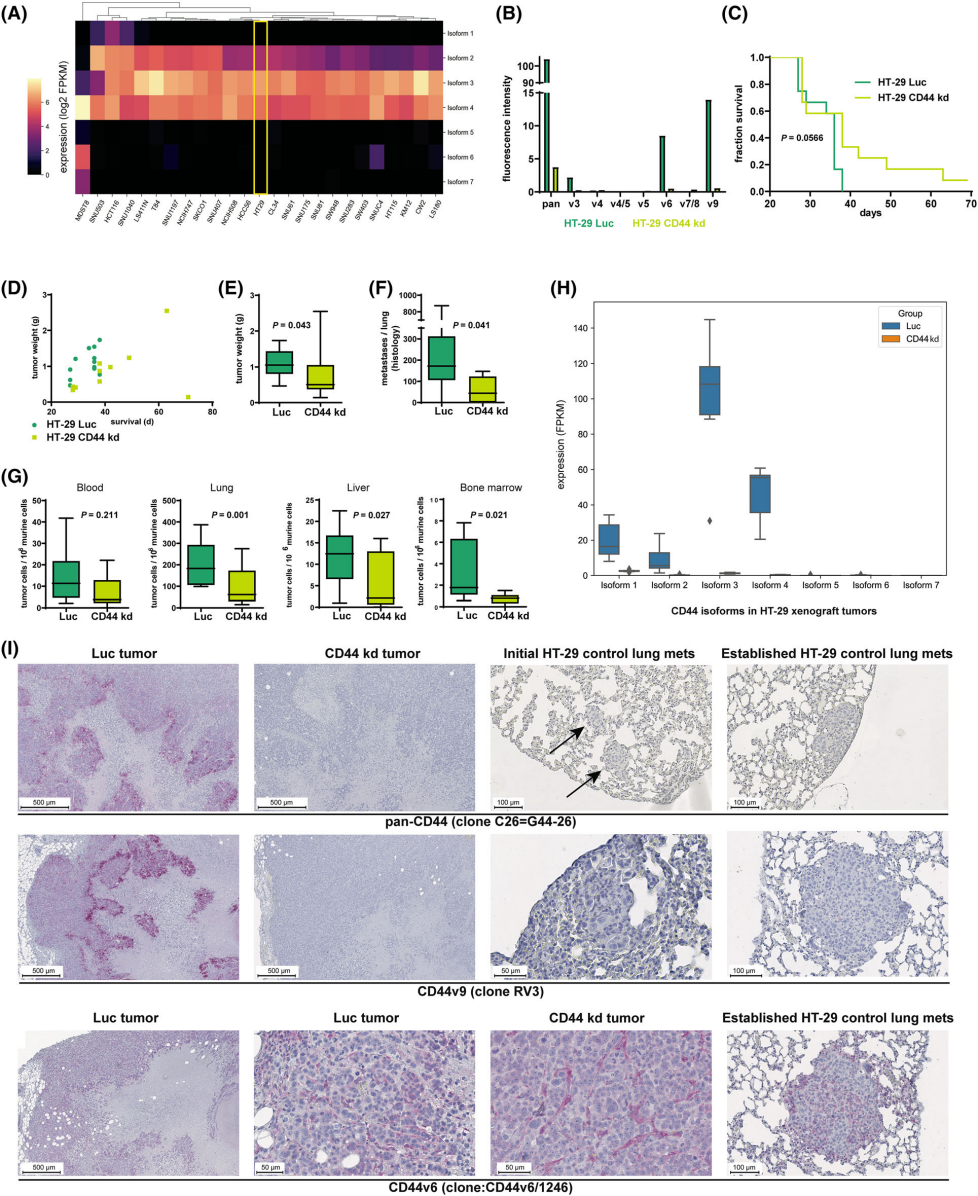
Pan‐CD44 kd results in decreased metastasis in HT‐29 xenograft models. (A) Heatmap of CD44 isoform 1–7 expression levels in commonly used human CRC cell lines. HT‐29 cells display strong CD44 isoform 3 and moderate CD44 isoform 4 expression. (B) Flow cytometric analysis of HT‐29 Luc (control shRNA, dark green) and CD44 kd (light green) cells for pan‐CD44 and CD44v levels as indicated (representative example is shown; *n* = 2). (C) Survival of mice after s. c. injection of HT‐29 Luc (*n* = 12) vs. CD44 kd (*n* = 12) cells (endpoint: s. c. tumor of ~ 1.5 cm^3^, skin ulceration above the tumor, or body weight change > 10%). (D) S. c. tumor weights at necropsy plotted against survival time across the two groups (*n* = 12). (E) Tumor weights at necropsy between the two groups (*n* = 12). (F) Lung metastasis numbers at necropsy between the two groups as determined by histology (*n* = 12 for HT‐29 Luc and *n* = 11 for CD44 kd). (G) Metastatic burden in distant organs/tissues: Blood (*n* = 12 for HT‐29 Luc and *n* = 11 for CD44 kd), Lung (*n* = 10 for HT‐29 Luc and *n* = 10 for CD44 kd), Liver (*n* = 10 for HT‐29 Luc and *n* = 11 for CD44 kd) and Bone marrow (*n* = 12 for HT‐29 Luc and *n* = 10 for CD44 kd) determined by *Alu*‐PCR across the two groups at necropsy. (H) CD44 isoform expression in HT‐29 Luc (*n* = 7) vs. CD44 kd (*n* = 7) xenograft tumors *in vivo*. (I) Immunostainings on tissues as indicated using a pan‐CD44 antibody, CD44v9‐specific antibody (isoforms 1, 2, and 3) and CD44v6‐specific antibody (isoforms 1 and 2). The arrows indicate spontaneous lung metastases of control xenografts. Note the lack of pan‐CD44 (upper row) and CD44v9 (middle row) reactivity in spontaneous lung metastases of control xenografts and in PTs of kd xenografts. CD44v6 is expressed in the murine tumor stroma of xenograft PTs and lung metastases and is not targeted by the CD44 kd (lower row), scale for the figures are at 500 μm, 100 μm, and 50 μm as indicated. The following statistical tests were used: log‐rank test (C), Wilcoxon rank sum test with continuity correction (E), multiple linear regression models adjusted for tumor weight (F, G).

Cell line database research considering the most commonly used human CRC cell lines revealed that HT‐29 cells represent the clinical *CD44* isoform abundance profiles quite well with strong *CD44* isoform 3 and moderate *CD44* isoform 4 expression (Fig. 3A). As previously shown by our group, HT‐29 xenografts metastasize spontaneously in a clinically relevant manner in s. c. xenograft models [81] and depend on E‐/P‐selectin for metastasis [17], suggesting a role of CD44 as a carrier of selectin ligands in this model. Therefore, the HT‐29 xenograft model was used in the present study as well.

CD44 was knocked down in HT‐29 cells using a pan‐CD44 shRNA construct. This construct stably reduced total CD44 expression by > 95% on the cell surface of vital HT‐29 cells as determined by flow cytometry (Fig. 3B). Commercially available, fluorescence‐labeled antibodies demonstrated expression of variant exons CD44v3, v6 and v9 on HT‐29 cells, indicating expression of isoforms 1 and/or 2 and/or 3 in these cells, all of which were abrogated upon pan‐CD44 kd. Antibodies against the products of variant exons v4, v4/5, v5, v7/8 revealed no detectable expression (Fig. 3B), but neither showed convincing signals in other cell lines and were, therefore, most likely not functional (data not shown).

To determine whether the CD44 kd has an influence on tumor growth and metastasis, HT‐29 Luc or CD44 kd cells were s. c. injected into SCID mice (*n* = 12), all of which developed s. c. PTs. All mice had to be euthanized due to tumor ulceration except two animals of the CD44 kd group. Of these two, one tumor reached the endpoint tumor weight (10% of the body weight), and the other animal had not reached any termination criteria when the experiment was stopped after 71 days. The survival of the mice (time from injection to termination criterion or endpoint) was by trend longer in the CD44 kd group (*P* = 0.057, log‐rank test, Fig. 3C). Fig. 3D further illustrates that control tumors reached higher tumor weights earlier than the CD44 kd tumors (by trend). The resulting tumor weights at necropsy were lower in the CD44 kd group (mean 0.7825 ± 0.654 g) than in the control group (mean 1.088 ± 0.384 g, *P* = 0.043, Wilcoxon rank sum test with continuity correction, Fig. 3E).

To quantify metastatic spread into the animals' organs, lung metastasis counts were determined by histology, and TC loads in blood, bone marrow, liver, and lung were determined by quantitative real‐time PCR for human *ALU* sequences (*ALU*‐PCR). In the statistical analyses, we calculated the *P*‐value for the effect of the CD44 kd on metastasis numbers while adjusting for the weight of the PT at necropsy. The histologically determined lung metastasis numbers were lower in the CD44 kd vs. control group (median 42, mean 56.3 ± 52.7 vs. median 172, mean 186.8 ± 70.9 metastases/lung, respectively; *P* = 0.041, adjusted regression, Fig. 3F). We did not observe differences in circulating TC counts in the animals' blood taken at necropsy (*P* = 0.211, adjusted regression, Fig. 3G). However, the metastatic burden in distant organs/tissues determined by *ALU*‐PCR was lower in the CD44 kd group compared with the control group (Fig. 3G, all values given are the median numbers of human TC/10^6^ murine cells; *P*‐values were calculated using linear regression adjusting for tumor weight): lung CD44 kd 67 vs. control 183 (*P* < 0.001); liver CD44 kd 2.1 vs. control 12.5 (*P* = 0.027); bone marrow CD44 kd 0.8 vs. control 1.8 (*P* = 0.021). We found a positive effect of the PT weight on liver metastasis counts [*P* < 0.001, regression coefficient: 8.85 (95% CI: 4.62–13.09)] and an almost significant positive effect on lung metastasis counts [*P* = 0.073, regression coefficient: 53.5 (95% CI: −5.51–112.62)] (not shown).

### 3.4 Pan‐CD44 kd mainly targets CD44 isoform 3 and 4 in HT‐29 xenografts *in vivo*

Based on bulk tissue RNA sequencing of HT‐29 xenograft PTs (harvested at necropsy), the pan‐CD44 kd sufficiently and stably targeted all detectable *CD44* isoforms *in vivo* (Fig. 3H). The most abundant isoforms were isoforms 3 and 4, whereas isoforms 1 and 2 were expressed at much lower levels; nevertheless, these two isoforms were also abrogated in the kd tumors. Isoforms 5–7 were not expressed (Fig. 3H). Aside from the qualitative similarity of *CD44* isoform expression pattern between HT‐29 cells *in vivo* (Fig. 3H) and *in vitro* (Fig. 3A), we also observed a high quantitative concordance. Specifically, the ratio of isoform 3 to isoform 4 was found to be 1.62 *in vitro* and *1.95 in vivo*. The slight relative increase in isoform 3 expression *in vivo* could be attributed to the more epithelial phenotype typically assumed for TCs that are re‐organizing as a 3D PT, in contrast to the more mesenchymal phenotype of 2D cultivated TCs. CD44 isoform 3 (CD44v) is recognized as a marker for a more epithelial phenotype, while CD44 isoform 4 (CD44s) is considered indicative of more mesenchymal cells [43].

Immunostainings using a pan‐CD44 antibody (recognizing an epitope in the product of an invariant standard exon) demonstrated induced expression of CD44 in the paranecrotic area of HT‐29 xenograft tumors. CD44 kd tumors were entirely negative in the pan‐CD44 staining (Fig. 3I, upper row). Importantly, histologically detectable spontaneous lung metastases (even though from the control group) showed no detectable CD44 staining with this antibody (Fig. 3I, upper row). We observed the same staining patterns with a CD44v9‐specific antibody (Fig. 3I, middle row), indicating putative expression of CD44 isoforms 1, 2 and 3. Using a CD44v6‐specific antibody, which specifically labels the products of CD44 isoforms 1 and 2, we observed immunoreaction of the murine tumor stroma only, which was also visible in the kd tumors and in lung metastases (Fig. 3I, lower row). Summarized, the pan‐CD44 staining (including isoform 4) and CD44v9 staining (indicating isoform 3) were co‐expressed in the paranecrotic area and absent in lung metastases, while the CD44v6 staining (indicating isoforms 1 and 2) was restricted to the PT stroma and metastasis stroma and was not affected by the kd.

### 3.5 Reduced metastasis upon CD44 kd is linked to partial EMT, angiogenesis‐ and mitochondria‐related pathways, as well as down‐regulation of CEACAM5

**Fig. 4:**
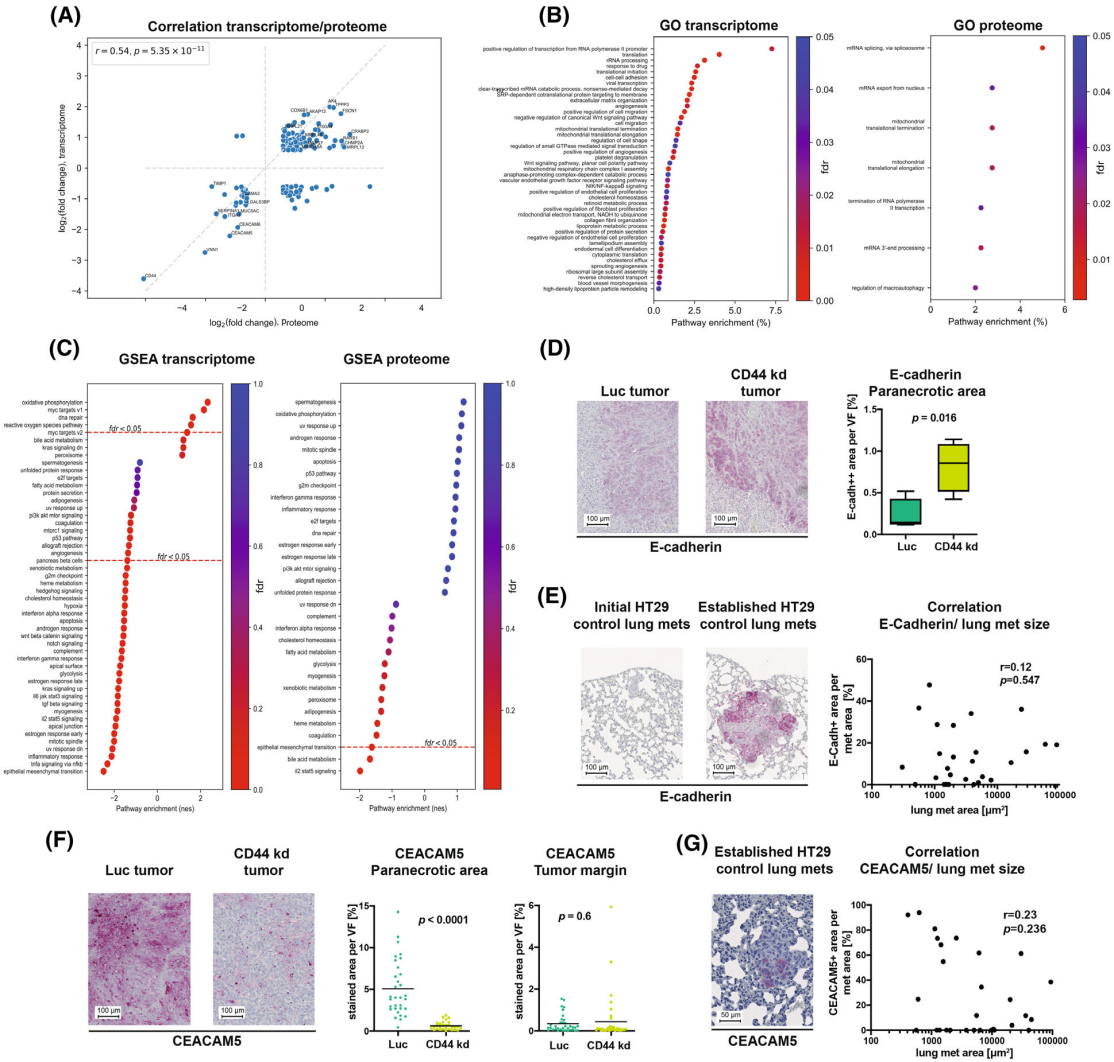
Transcriptome and proteome profiling of HT‐29 xenograft tumors upon pan‐CD44 kd. (A) Correlation of transcriptome and proteome changes detectable in HT‐29 xenograft PTs upon pan‐CD44 kd (*n* = 130 mRNAs/proteins). (B) GO pathway enrichment analyses of mRNAs and proteins differentially expressed in HT‐29 Luc vs. CD44 kd xenograft tumors. Note the significant alteration of mitochondrial translational elongation and termination in both transcriptome and proteome analyses. (C) GSEA of the same datasets shows down‐regulation of the EMT program in the less metastatic CD44 kd xenografts. (D) Percentage of PT cells with enhanced E‐cadherin expression (E‐cadh++) in paranecrotic areas of CD44 Luc vs. kd xenografts (*n* = 4), scale = 100 μm. (E) Correlation between the E‐cadherin staining intensity and the respective size of spontaneous lung metastases of HT‐29 control xenografts (*n* = 27), scale = 100 μm. (F) CEACAM5 immunoreactivity in the paranecrotic areas vs. tumor margins of HT‐29 Luc vs. CD44 kd xenografts and results of the quantification within the paranecrotic area (*n* = 32 for HT‐29 Luc and *n* = 29 for CD44 kd), scale = 100 μm. (G) CEACAM5 staining in spontaneous lung metastases of HT‐29 control xenografts and correlation between CEACAM5 staining intensity and size of the respective lung metastases (*n* = 29), scale = 50 μm. Black lines (F) indicate median values. The following statistical tests were used: Spearman's correlation test (A, E, G), unpaired Student's *t*‐test (D, F).

To clarify the possible mechanisms underlying decreased metastasis numbers, we performed bulk RNA sequencing and proteomic analysis of HT‐29 control vs. CD44 kd tumors. A total of 4214 protein‐coding genes showed significant expression alteration upon CD44 kd according to RNA‐seq data (fold changes were at least 1.5 up‐ or down‐regulated, FDR < 0.05). Proteome analysis identified a total of 2403 human proteins, 415 of which were differentially expressed. The intersection of differentially expressed mRNAs and proteins contained 130 entries; notably, fold changes in transcriptomic and proteomic analyses were strongly correlated (*r* = 0.54, *P* = 5.35 × 10^−11^, Fig. 4A, see Table S5 for all overlapping molecules). This intersection included significant down‐regulation of, as expected, CD44 and further “mesenchymal” or pro‐migratory molecules such as TIMP1 [82], ITGA1, LAMA3, SERPINA1 [83], but also of “epithelial” molecules such as LGALS3BP [84], MUC5AC and stem cell markers such as CEACAM5/6 and ITGA6 in the less metastatic CD44 kd xenografts (further stem cell markers such as *CD24*, *PROM1* and *KLF4* were found down‐regulated by RNA‐seq only). Conversely, molecules such as TPPP3, FSCN1, CRABP2, COX6B1, AKAP12, S100A4, RARS1, CHMP2A and several mitochondria‐related proteins, such as AK4, MRPL12/21/46 and MRPS7/18A were up‐regulated in the less metastatic CD44 kd tumors based on transcriptome and proteome analyses. RNA‐seq‐based fold changes for key genes were validated by RT‐qPCR (Fig. S3).

Gene Ontology pathway enrichment analyses of differentially expressed mRNAs and proteins revealed deregulation in multiple cellular pathways (Fig. 4B). Of note, we observed significant enrichment of mitochondria‐related pathways in both transcriptome and proteome data (e.g., mitochondrial translational elongation and termination). In the transcriptome data, we further identified significant regulation of pathways related to angiogenesis (GO terms angiogenesis, positive regulation of angiogenesis, vascular endothelial growth factor (VEGF) receptor signaling pathway, positive and negative regulation of EC proliferation, sprouting angiogenesis, blood vessel morphogenesis), cell migration, adhesion, and ECM organization. Based on GSEA, we observed strong down‐regulation of the EMT program (Fig. 4C), including a decrease of several well‐known mesenchymal markers (TIMP1, TGFBI, ITGA2) in the less metastatic CD44 kd xenografts. Interestingly, expression of the canonical epithelial marker E‐cadherin (*CDH1*) was two‐fold decreased in RNA‐seq data, while a slight increase in E‐cadherin protein expression was captured by proteome analysis (1.2‐folds, adjusted *P* = 0.00454). Classical EMT transcription factors (*SNAI1*, *SNAI2*, *ZEB1*, *ZEB2*, *TWIST*) were expressed at very low levels by HT‐29 xenografts according to the RNA‐seq data.

As the GSEA nevertheless suggested the involvement of the EMT program in conjunction with the CD44 kd‐mediated reduction in metastasis numbers, we stained HT‐29 xenograft PTs as well as spontaneous initial and established lung metastases for E‐cadherin and vimentin. Of note, the less metastatic CD44 kd PTs indeed showed a significant increase (*P* = 0.016, unpaired *t*‐test) in strongly E‐cadherin‐positive (E‐cadh++) TCs in the paranecrotic area (Fig. 4D), where CD44 had been found to be induced in control tumors (see above, Fig. 3I). However, there was no correlation between the E‐cadherin staining intensity and the respective size of lung metastases (*r* = 0.12, Fig. 4E), indicating that initial metastatic cell clusters did not reliably show less E‐cadherin expression than established metastases in the HT‐29 xenograft model. Likewise, vimentin staining, which was successfully established on control tissues, did not show staining in either the initial or established lung metastases (Fig. S4A). We, therefore, concluded that the regulation of EMT‐related molecules as per “omics” analyses rather reflected a partial switch between E and M states during metastasis in the used model.

We then aimed to validate the regulation of CEACAM5/6, which were down‐regulated according to both transcriptomics and proteomics analyses (see above, Fig. 4A). Interestingly, CD44 kd tumors showed nearly abolished CEACAM5 immunoreactivity in the paranecrotic areas (again, the area where CD44 was expressed in control tumors, see Fig. 3I) compared to control tumors (*P* < 0.0001, unpaired *t*‐test), but not at the tumor margin (*P* = 0.6, Fig. 4F). CEACAM5 was also detectable in lung metastases, but there was no significant correlation between CEACAM5 staining intensity and size of the respective lung metastases (*r* = 0.23, Fig. 4G). CEACAM6 levels did not differ between control and CD44 kd PTs (Fig. S4B). Furthermore, we aimed to validate the findings related to ECM re‐organization as suggested by the “omics” data but did not find obvious changes in the content or distribution of HA or connective tissue in control vs. CD44 kd PTs by means of histological analyses (Fig. S4C,D).

### 3.6 Reduced metastasis upon CD44 kd is linked to enhanced KDR signaling and microvessel number, less hypoxia and higher mitochondrial content in the paranecrotic tumor area

**Fig. 5:**
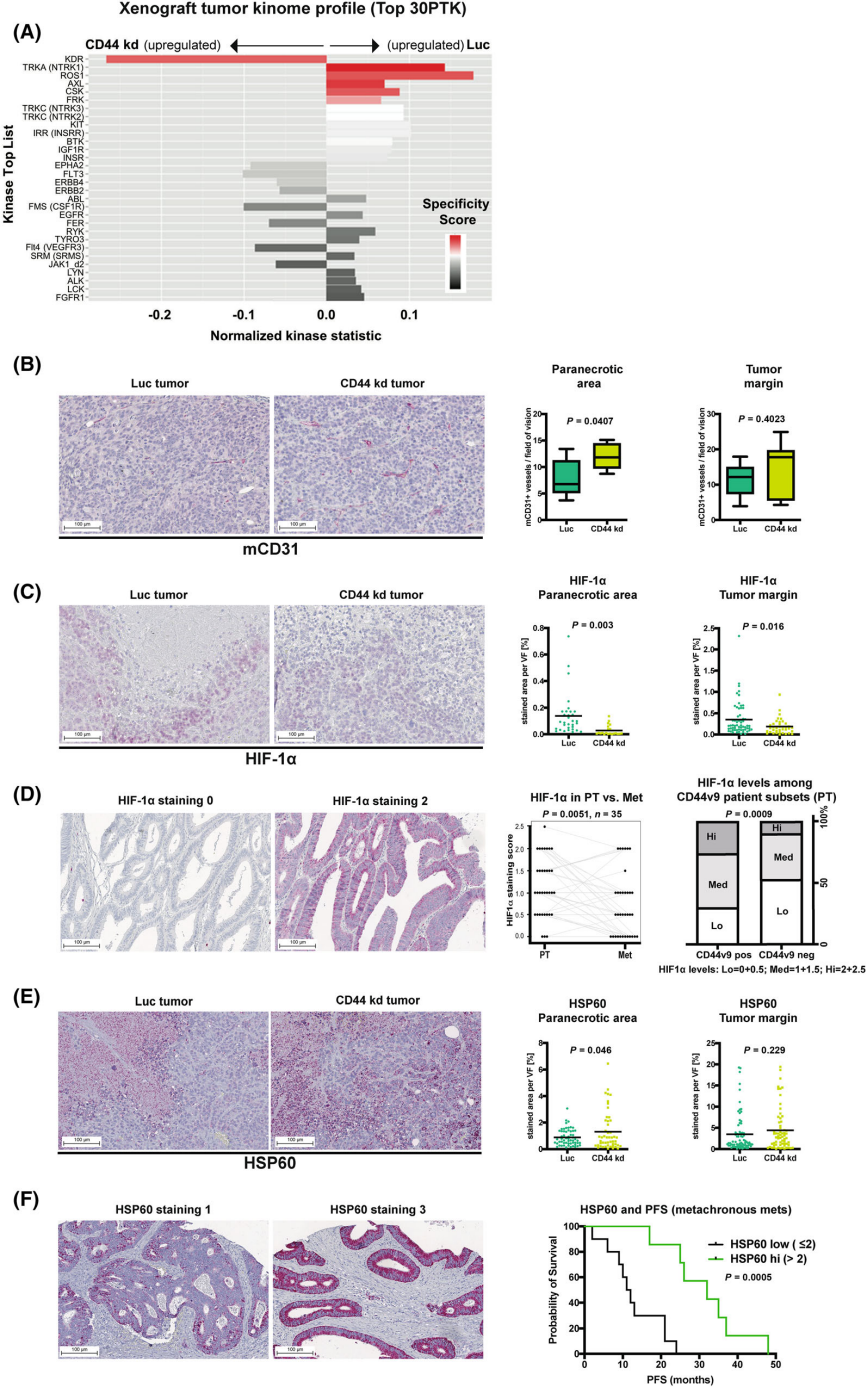
Kinomic profiling, microvessel density, hypoxia and mitochondrial content in HT‐29 xenograft tumors upon pan‐CD44 kd. (A) Kinomic profiling of phospho‐tyrosine kinase (PTK) activity in HT‐29 Luc vs. CD44 kd xenograft PTs. Note the specific up‐regulation of KDR (VEGFR‐2) signaling in the less metastatic CD44 kd xenograft tumors (*n* = 4). (B) Anti‐mCD31 immunostaining indicates the number of tumor microvessels in the paranecrotic areas and at the margins of HT‐29 Luc vs. CD44 kd tumors (*n* = 6), scale = 100 μm. (C) HIF‐1α levels were determined by immunohistochemistry in the paranecrotic areas (*n* = 32 for HT‐29 Luc and *n* = 21 for CD44 kd) and at the margin (*n* = 62 for HT‐29 Luc and *n* = 41 for CD44 kd) of xenograft tumors of the two groups, scale = 100 μm. (D) HIF‐1α levels in matched pairs of PTs and liver metastases (Met) from CRC patients. HIF‐1α PT levels among CD44v9‐negative vs. CD44v9‐positive patients (see Fig. 2D also), scale = 100 μm. (E) HSP60 staining indicates the mitochondrial content in the paranecrotic areas (*n* = 59 for HT‐29 Luc and *n* = 50 for CD44 kd) and at the margins (*n* = 77 for HT‐29 Luc and *n* = 65 for CD44 kd) of HT‐29 Luc vs. CD44 kd xenograft tumors, scale = 100 μm. (F) PFS (time from PT surgery to occurrence of metachronous liver metastases) of CRC patients with high vs. low HSP60 PT expression, scale = 100 μm. Two slides on the left show representative examples of HSP60 low (score 1) and high (score 3) tumors. Black lines in (C) and (E) indicate median values. The following statistical tests were used: unpaired Student's *t*‐test (B, C, E), paired Student's *t*‐test (“HIF‐1α in PT vs. Met” panel in D), Pearson's chi‐square test (“HIF‐1α levels among CD44v9 patient subsets (PT)” panel in D), log‐rank test (F).

Based on kinomics profiling considering phospho‐tyrosine kinases (PTK) and subsequent upstream analysis, we identified specific up‐regulation of KDR (VEGFR‐2) signaling in the less metastatic CD44 kd xenograft tumors (Fig. 5A). Since the increase in KDR (VEGFR‐2) signaling matched very well with the altered angiogenesis pathways determined by transcriptomics (see above), we further investigated the number of microvessels per viewing field in HT‐29 control vs. CD44 kd xenograft tumors using anti‐mCD31 immunostaining. The number of tumor microvessels was significantly increased in the paranecrotic areas of CD44 kd tumors (*P* = 0.0407, unpaired *t*‐test) but not at the tumor margin (Fig. 5B). We then analyzed hypoxia induction by means of immunostainings for HIF‐1α and HIF‐2α. We observed that HIF‐1α was enhanced in the paranecrotic areas of control tumors and significantly decreased upon CD44 kd (*P* = 0.003, unpaired *t*‐test, Fig. 5C). HIF‐2α was widely expressed in the vital tumor masses between the tumor margin and necrotic center but was not altered upon CD44 kd (Fig. S4E). To test whether the suggested link between metastasis formation, hypoxia and CD44 (most likely isoform 3, as this was the most abundant isoform, see above) could also be established in clinical samples, we stained consecutive slides of primary CRC specimens and matched liver metastases for HIF‐1α and CD44v9 (isoform 3 contains v8‐v10). Indeed, the HIF‐1α staining score was significantly lower in liver metastases compared to matched PTs (*P* = 0.0051, paired *t*‐test, Fig. 5D). Moreover, HIF‐1α levels in patient PTs were highly significantly diminished in the subgroup of CD44v9‐negative compared with CD44v9‐positive patients (*P* = 0.0009, *χ* ^2^ test, Fig. 5D), which was very consistent with the decrease in hypoxia after CD44 kd *in vivo*.

These and the mitochondria‐related findings (Fig. 4A–C) suggested that CD44 might affect oxygenation and, thus, mitochondrial metabolism. Therefore, we re‐analyzed our transcriptomics data, focusing on the OxPhos process. OxPhos of glucose takes place in mitochondria in the presence of oxygen to provide energy (ATP molecules) to cells. Interestingly, the expression of almost all genes from the OxPhos gene set (52 out of 60 genes) was increased upon CD44 kd (Table S6). Therefore, we determined the mitochondrial content in the xenograft PTs using anti‐HSP60 staining and revealed a significant increase in the HSP60‐stained area in the paranecrotic area of the less metastatic CD44 kd xenografts (*P* = 0.046, unpaired *t*‐test, Fig. 5E). Accordingly, CRC patients with high PT HSP60 levels had a significantly improved PFS compared with patients with low HSP60 levels (median survival HSP60 low: 11.5 months; HSP60 high: 32 months; *P* = 0.0005 Log‐rank (Mantel‐Cox) test, Fig. 5F).

### 3.7 VEGF release and EC tube formation are reduced after CD44 kd in normoxia but exorbitantly increased in hypoxia

**Fig. 6:**
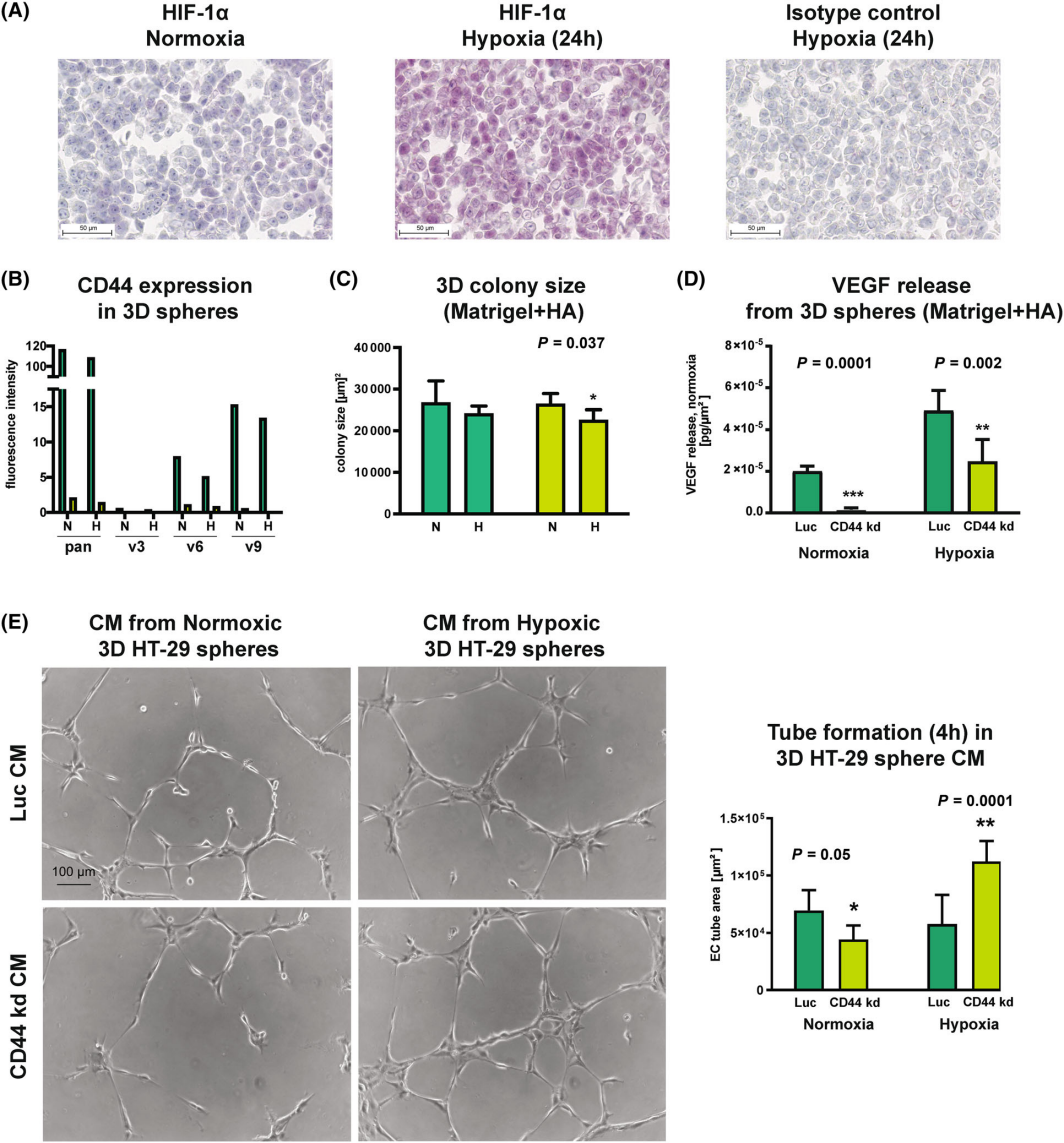
Colony‐forming capacity of and VEGF release from HT‐29 spheres in normoxia vs. hypoxia upon CD44 kd. (A) Validation of the used HIF1α immunostaining protocol using HT‐29 cells cultivated under normoxia or hypoxia (representative example is shown; *n* = 2), scale = 50 μm. (B) Flow cytometric analysis of pan‐CD44 and CD44v expression on HT‐29 cells recovered from 3D spheres cultured in normoxia (N) vs. hypoxia (H) (representative example is shown; *n* = 2). (C) 3D colony‐forming ability of CD44 kd compared to control cells in N vs. H in Matrigel containing HA. *P = 0.037 in (C) refers to CD44 kd H vs. CD44 kd N (*n* = 5). (D) VEGF release per colony area from 3D control vs. CD44 kd HT‐29 spheres grown in Matrigel containing HA under normoxic vs. hypoxic conditions (*n* = 5). ***P = 0.0001 in (D) refers to CD44 kd normoxia vs. Luc normoxia; **P = 0.002 in (D) refers to CD44 kd hypoxia vs. Luc hypoxia (E) EC tube formation over 4 h on growth factor‐reduced Matrigel in the presence of CM harvested from 3D control vs. CD44 kd HT‐29 spheres grown in Matrigel containing HA under normoxic vs. hypoxic conditions (*n* = 5), scale = 100 μm. *P = 0.05 in (E) refers to CD44 kd normoxia vs. Luc normoxia; **P = 0.0001 in (E) refers to CD44 kd hypoxia vs. CD44 kd normoxia. Error bars represent standard deviation. Unpaired Student's *t*‐test was used in C, D, E.

To further investigate the link between CD44, hypoxia and angiogenesis, HT‐29 cells were first cultivated on poly‐HEMA‐coated plastic to form spheres under normoxic or hypoxic conditions. Hypoxia induction was validated after 24 h using anti‐HIF1α (Fig. 6A) and anti‐HIF‐2α (Fig. S4E) immunohistochemistry as described above. The induced expression of pan‐CD44 and CD44v9 observed in the paranecrotic areas of xenograft tumors (Fig. 3I) could not be mimicked by hypoxic cultivation of HT‐29 3D spheres in poly‐HEMA‐coated flasks (Fig. 6B). Under more sophisticated 3D conditions in a matrigel‐ and HA‐containing environment, we observed a marginal but significant negative effect of hypoxia on the colony‐forming ability of CD44 kd but not control cells on day nine after seeding (*P* = 0.037, unpaired *t*‐test, Fig. 6C). Under normoxic conditions, the 3D spheres of the CD44 kd group released significantly less VEGF per colony area than those from the control group (CD44 kd: 1.53 × 10^−6^ pg/μm^2^; control: 2.0 × 10^−5^ pg/μm^2^, *P* < 0.0001, unpaired *t*‐test, Fig. 6D, left diagram). However, the transfer of the 3D spheres to hypoxic conditions in CD44 kd resulted in a much greater increase in VEGF release (by factor + 16.1) than in the control (by factor + 2.45, *p* = 0.002, unpaired *t*‐test, Fig. 6D, right diagram).

Aside from VEGF release quantification, we used EC tube formation assays to profile differences in angiogenesis in the presence of CM from CD44 kd or control 3D HT‐29 spheres cultivated under normoxic or hypoxic conditions (Fig. 6E). Concordantly with the VEGF release, EC tube formation was slightly reduced in CM from CD44 kd spheres under normoxic conditions (*P* < 0.05, Fig. 6E). However, the CM from CD44 kd spheres generated under hypoxic conditions strongly increased tube formation (*P* = 0.0001, Fig. 6E).

Further conventional (2D) *in vitro* assays for tumor growth‐ and metastasis‐related TC features revealed inconsistent results, which were only partially in line with the *in vivo* observations: upon CD44 kd, HT‐29 TC proliferation was increasingly improved over time (Fig. S5A), cell migration was enhanced (Fig. S5B), and invasion was decreased (Fig. S5C). Unexpectedly, the CD44 kd did not decrease the expression of the canonical E‐selectin ligand SLe^A^ but even significantly improved E‐selectin binding capacity under static incubation conditions (*P* = 0.0093, unpaired *t*‐test, Fig. S5D,E). However, the adhesive behavior of the TCs in a laminar flow adhesion assay on HUVEC was not significantly altered under these conditions (*P* = 0.1, Fig. S5F).

### 3.8 Transcriptomes of patient samples with low *CD44* isoform 4 expression overlap with the transcriptomic signature of pan‐CD44 kd xenografts

While pan‐CD44 kd in the xenograft model resulted in decreased metastasis, our initial analysis of left CRC samples suggested that *CD44* isoform 3 and isoform 4 were oppositely correlated with the OAS of patients (see above). From the two isoforms, only isoform 4 “matched” with the phenotype of pan‐CD44 kd HT‐29 cells; specifically, low isoform 4 expression was associated with a better prognosis (Fig. 2B).

**Fig. 7:**
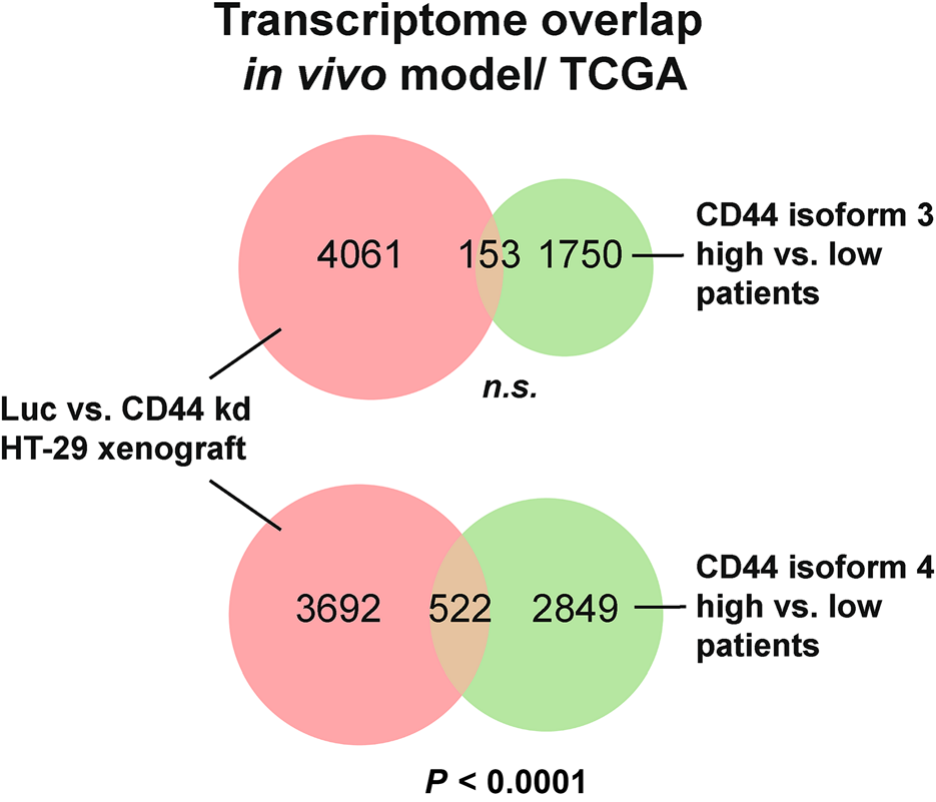
Comparison of transcriptomes between HT‐29 xenograft tumors and PTs of colon cancer patients (TCGA). Transcriptome overlap between genes differentially expressed among CD44 isoform 3 (upper panel) or isoform 4 (lower panel) high vs. low left colon cancer patients (TCGA) and genes differentially expressed between HT‐29 Luc vs. CD44 kd xenograft PTs. Note the significant overlap between isoform 4 (but not isoform 3) high vs. low patients and *in vivo* data. The permutation test was used to estimate the *P*‐value.

Therefore, we next compared the transcriptome differences detected in control vs. CD44 kd HT‐29 xenograft tumors with TCGA transcriptome differences between *CD44* isoform 4 high vs. low left CRC patients. Strikingly, the two sets of DEGs overlapped significantly and concordantly (*P* < 0.0001, Fig. 7). This association could not be observed for the transcriptome differences between *CD44* isoform 3 high vs. low left CRC patients (Fig. 7). Indeed, these results could also be observed on the level of pathway analysis: as mentioned above, low *CD44* isoform 4 (but not isoform 3) expression in patients was associated with less EMT and more OxPhos (Fig. 2F,G), mirroring the phenotype of pan‐CD44 kd HT‐29 xenografts. Therefore, the functional *in vivo* effects observed in our experiments were most probably due to a reduction of the second most abundant isoform, i.e., isoform 4, achieved by the pan‐CD44 kd.

## 4 Discussion

CD44 functions as a glycoreceptor for HA and further ECM components, is involved in matrix degradation, carries glycoligands for E‐, P‐ and L‐selectins, and determines EMT and CSC states in several cancers [17, 32, 42, 85]. Therefore, CD44 has attracted great interest in metastasis research over the past decades [31, 38, 46, 80, 86, 87, 88, 89]. However, a number of published studies have reported conflicting results, presumably because they were based on very different approaches, such as *in vitro*‐only studies [90], syngeneic or genetically engineered models [86]. The experimental metastasis xenograft models usually involved the injection of TCs directly into the bloodstream [88, 89], bypassing the early steps of the metastatic cascade that occur in PTs. Direct evidence from spontaneous metastasis xenograft models, which include PT growth, is relatively rare [91, 92], particularly in CRC. CD44 research is further complicated by the ambiguous and inconsistent nomenclature of CD44 isoforms [42]. The designation of single variant exons is sometimes confused with the designation of isoforms, although certain variant exons are contained in multiple isoforms. Reliable antibodies are not available for all variant exons, which might explain why previous research focused on CD44v3, v6, and v9. We also tested commercial antibodies against v4, v4/5, v5, v7/8 here and found no convincing reactivity towards cell lines from several tumor types (data not shown). Hence, well‐designed studies are needed to investigate the roles of CD44 isoforms in cancer.

To clarify the significance of CD44 isoforms in CRC, we first identified that *CD44* isoforms 3 and 4 (according to NCBI nomenclature) are the most abundant isoforms in CRC. Isoform 3 is up‐regulated in cancer vs. normal mucosa, and the HT‐29 cell line, which we knew to metastasize spontaneously in xenograft models in an E‐/P‐selectin‐dependent manner [17], resembles the expression of the clinically relevant isoforms. Using a pan‐CD44 kd approach, we obtained direct *in vivo* evidence that CD44 promotes tumor growth and spontaneous distant metastasis to multiple sites in the HT‐29 xenograft model. The effect of the CD44 kd on metastasis numbers was statistically independent of its concurrent effect on the PT. The pan‐CD44 kd largely affected isoforms 3 and 4, which were the most abundant ones in the xenograft PTs. Pan‐CD44 and CD44v9 antibodies, which recognize all isoforms and isoforms 1–3, respectively, showed induced immunoreactivity in the paranecrotic areas. CD44v6 (present in isoforms 1 and 2) was specifically detectable in the murine stroma but not in the TCs themselves, and was not affected by the CD44 kd as determined by immunohistochemistry. Therefore, the observed anti‐metastatic effect of pan‐CD44 shRNA was most likely mediated via a reduction of CD44 isoforms 3 and 4. Importantly, however, neither of these isoforms was convincingly detectable on metastatic TCs. Thus, we concluded that the anti‐metastatic effect was exerted at the PT site. Accordingly, the CD44 kd did not impair but rather improved the adhesive properties of HT‐29 cells (E‐selectin binding, SLe^A^ expression), contrasting our previous observations with melanoma and osteosarcoma cells [41]. Thus, different CD44 isoforms might mediate metastasis formation in the case of these (non‐epithelial) tumors.

**Fig. 8:**
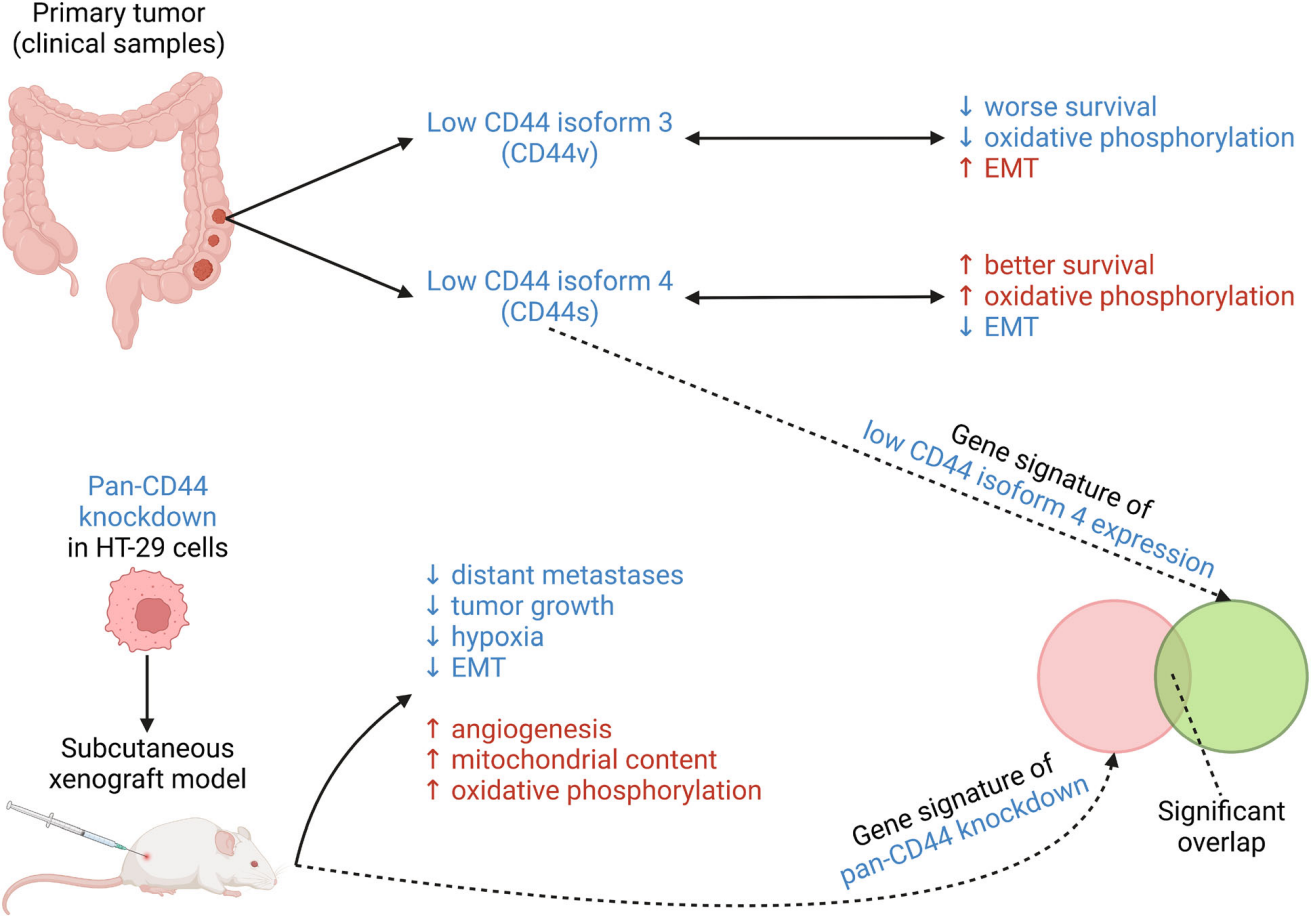
Graphical model of the main findings of our study. Top part: low expression of CD44 isoform 4 (but not isoform 3) correlates with better patient outcomes, increased OxPhos and reduced EMT in CRC PTs (TCGA). Bottom part: pan‐CD44 kd decreases spontaneous metastasis in human CRC xenograft models. Intratumoral gene expression alterations upon kd significantly overlap with genes differentially regulated among CD44 isoform 4 (but not isoform 3) high vs. low patients. The corresponding gene sets and pathways in xenograft models include EMT, angiogenesis, and OxPhos. The Figure was created with BioRender.com.

Unexpectedly, the prognostic significance of CD44v9 in patients was opposite to the functional role of CD44 *in vivo*. In this respect, the question arose whether the functional effects of the CD44 kd were a direct consequence of a reduction of the most abundant isoform 3. TCGA analyses confirmed that a higher *CD44* isoform 3 level was associated with improved OAS. In contrast, a higher *CD44* isoform 4 level indicated a shortened survival. Consistent with this opposing prognostic relevance of the *CD44* isoforms, the genes regulated by *CD44* isoform 3 or 4 in patients were, to a large extent, identical but almost exclusively oppositely regulated. Importantly, the transcriptomic landscape of CD44 kd xenografts showed a significant, concordant overlap with the genes regulated by *CD44* isoform 4 but not isoform 3 in patients. Deduced from this finding, we suggest that the functional effects of our pan‐CD44 kd were most likely due to the regulation of the second most abundant isoform, i.e., isoform 4. Whether or not CD44 isoform 4 can be specifically targeted (without altering other CD44 isoforms) and if such an approach also leads to reduced metastasis of HT‐29 xenografts *in vivo* will be the basis of a substantial future study. We summarized the study results in a simple graphical model – see Fig. 8.

Despite the demonstrated pro‐metastatic role of CD44 isoform 4, we also showed that the expression of this molecule is higher in adjacent normal tissues compared to tumor samples (Fig. 1B). Of note, a pro‐metastatic and pro‐mesenchymal role of CD44 isoform 4 was previously shown in other cancer types, including breast cancer [45, 47, 93], hepatocellular carcinoma [94] and squamous cell carcinoma [95]. One of the conjectures that can explain such phenomena is possibly a “non‐linear” role that isoform 4 plays in the different steps of cancer development. For example, switching from isoform 4 to isoform 3 could be beneficial during malignant transformation, while higher isoform 4 expression could give an advantage for metastasizing cancer cells. Deciphering such an interplay between CD44 isoforms in the course of CRC development and progression remains one of the most intriguing open problems.

The induced expression of CD44 in the paranecrotic area of HT‐29 control xenograft tumors (compared to tumor margin) indicates a functional link between CD44 and tumor hypoxia response [96, 97, 98, 99]. Therefore, it was particularly interesting to observe decreased hypoxia in the paranecrotic areas of CD44 kd tumors, accompanied by decreased EMT and enhanced OxPhos in whole CD44 kd tumors (based on RNA‐seq and proteome data) and in its paranecrotic area in particular (based on immunostainings). These findings suggest that not only hypoxia and HIF‐1a activate CD44 expression, but CD44, in turn, affects hypoxia. The paranecrotic regions of the HT‐29 xenograft PTs showed induced expression of HIF‐1α, demonstrating hypoxic conditions in those areas where pan‐CD44 and CD44v9 were induced under control conditions. Such an association between hypoxia and CD44 induction is supported by previous studies that demonstrated HIF‐1α transcriptional activation of CD44 in nucleus pulposus cells [100] and co‐localization of hypoxia and CD44 expression in breast cancer xenografts [97]. Transcriptomics, proteomics, and kinomics analyses, as well as a number of subsequent validation steps, collectively demonstrated that the reduction in metastasis upon CD44 kd was accompanied by improved angiogenesis, reduced hypoxia, decreased (partial) EMT, enhanced production of mitochondrial proteins, and induced mitochondrial OxPhos in the xenograft PTs. In our mechanistic studies, in a matrigel/HA‐containing 3D environment, CD44 kd spheres secreted significantly less VEGF than control spheres under normoxic conditions, while VEGF release was markedly increased from CD44 kd spheres by hypoxic conditions. This enhanced VEGF release under hypoxic conditions fits well with the enhanced microvessel formation in the paranecrotic regions of CD44 kd tumors, leading to reduced HIF‐1α levels in the microenvironment. These findings do not support the possibly too simplistic assumption that more tumor microvessels automatically cause more metastases. Instead, vessel normalization and, thereby, improved oxygenation have been shown to reduce metastasis [101]. Our findings support the concept that hypoxia is a key driver of metastasis due to its role in promoting EMT via the HIFs [5, 6, 102]. Accordingly, EMT was one of the most strongly down‐regulated pathways upon CD44 kd, reflected by increased E‐cadherin levels in the paranecrotic regions of CD44 kd tumors. Along these lines, EMT was strongly enriched in patients with high *CD44* isoform 4 expression in the TCGA database. However, the size of lung metastases in our *in vivo* model did not correlate with their respective E‐cadherin level, and HT‐29 xenografts lacked expression of vimentin and classical EMT transcription factors (*SNAI1*, *SNAI2*, *ZEB1*, *ZEB2*, *TWIST*). Therefore, the HT‐29 model apparently represents one of the intermediate EMT phenotypes where one would expect metastasis to be particularly altered by a change in the E/M status [15].

While the role of endothelial CD44 for angiogenesis is well described [85, 103, 104, 105, 106, 107], a direct influence of TC CD44 on VEGF release and, thus, tumor microvessel formation has so far, to the best of our knowledge, rarely been described. In a study related to head and neck squamous cell cancer, CD44+ TCs contained pro‐angiogenic factors and stimulated tumor angiogenesis in comparison to CD44‐ TCs [87], which fully supports our observation of decreased VEGF release by CD44 kd spheres in normoxia. However, it remains to be determined how the more pronounced enhancement of VEGF release under hypoxic conditions specifically in CD44 kd spheres has occurred.

The improved microvessel formation in CD44 kd tumors was linked to reduced (partial) EMT, enhanced HSP60 protein expression levels (indicating the presence of mitochondria), and up‐regulated mitochondrial OxPhos. Intriguingly, previous reports by others also showed that a down‐regulation of mitochondrial genes is associated with both EMT induction and poor clinical outcomes across a range of tumor types [108]. The latter observation is directly supported by our finding of decreased PFS of HSP60 low patients. Moreover, it has been suggested that EMT enhances glycolysis (the metabolic “counterpart” of mitochondrial OxPhos in TCs) and confers TCs with CSC‐like characteristics [109]. Accordingly, the kd of the CSC marker CD44 in the present study was not only accompanied by decreased EMT and increased OxPhos, but also by a strong decrease in the CSC marker CEACAM5, a gene encoding the cell surface glycoprotein that represents the founding member of the carcinoembryonic antigen (CEA) family of proteins. It remains to be determined whether CD44 directly regulates CEACAM5 expression or whether the loss of CEACAM5 is a concomitant sign of the generally reduced stem‐like properties after CD44 kd. Intriguingly, ablation of CD44 has already been linked with a metabolic shift from glycolysis to OxPhos in breast cancer cells [110].

One of the main limitations of the present study is the use of pan‐CD44 kd shRNA constructs. Our analyses of clinical tumor samples suggest that CD44 isoforms 3 and 4 play opposing roles in CRC metastasis. To provide a more nuanced understanding of these findings, experimental perturbations targeting individual isoforms are required. Another related limitation of our study is the use of a single anti‐CD44 shRNA construct. However, we could largely rule out major off‐target effects using high‐throughput transcriptome and proteome analyses. Directions for future research should include the development of isoform‐specific kd constructs. Additionally, pan‐*CD44* CRISPR knockout experiments followed by the overexpression of distinct isoforms would be valuable in deepening our mechanistic understanding of these associations both *in vitro* and *in vivo*.

## 5 Conclusions

In summary, we provide direct evidence that CD44 promotes spontaneous distant metastasis in a CRC xenograft model through PT‐related effects. Although isoform 3 is more abundantly expressed, the transcriptomic landscape achieved by the CD44 kd specifically overlaps with the transcriptome differences between *CD44* isoform 4 high vs. low left‐sided CRC patients. Accordingly, the prognostic relevance of *CD44* isoform 4 for patients with left‐sided CRC specifically reflects the pro‐metastatic role of CD44 observed *in vivo*. Both CD44 isoforms are induced in the paranecrotic area of xenograft tumors, where enhanced levels of HIF‐1α can be detected. Hypoxic conditions much more strikingly enhance VEGF release from CD44 kd as compared to control spheres, explaining the improved angiogenesis in the paranecrotic region of CD44 kd xenograft tumors. Furthermore, depletion of CD44 reduces the intermediate E/M phenotype and stem cell properties in HT‐29 xenografts, accompanied by enhanced mitochondrial content and OxPhos. These findings demonstrate a decisive influence of CD44 isoform 4 on metastasis by regulating tumor angiogenesis under hypoxic conditions. The obtained reduction in hypoxia, EMT, and improved mitochondrial metabolism limit the metastatic propensity of the tested CRC xenografts. Our findings suggest CD44 isoform 4 as a potential drug target for the discovery of clinically useful inhibitors of hematogenous metastasis formation in CRC.

## Conflict of interest

The authors declare no conflict of interest.

## Author contributions

Conceptualization: AE‐D, SN, DM, AT, US, DW, TL. Data curation: AE‐D, SN, TL. Investigation: AE‐D, SN, DM, HM, VN, JS‐S, VF, JLS, MCB, AS, M‐TH, MK, OE, HB, L‐CC, MR, LK. Methodology: AE‐D, SN, DM, MvI, AT, US, DW, TL. Software: AE‐D, SN, VN. Writing – original draft: AE‐D, SN, DM, US, DW, TL.

## Supporting information


**Fig. S1.** Dependence of *CD44* isoforms' expression levels on colon cancer stage and isoform‐isoform correlations (TCGA).

**Fig. S2.** *CD44* isoforms 1–7 expression in left and right‐sided colon cancer tissues (TCGA).

**Fig. S3.** Validation of RNA‐Seq data by RT‐qPCR.

**Fig. S4.** Expression of selected proteins in HT‐29 xenograft primary tumors, lung metastases and in HT‐29 cells under normoxic vs. hypoxic conditions.

**Fig. S5.** 2D cell culture‐based *in vitro* assays for tumor growth‐ and metastasis‐related tumor cell features.

**Table S1.** Summary of immunohistochemical staining protocols relevant to this study.

**Table S2.** Primer sequences and PCR efficiencies of all primer sets used in this study.

**Table S3.** Differentially expressed genes between CD44 isoform 3 high vs. low patients.

**Table S4.** Differentially expressed genes between CD44 isoform 4 high vs. low patients.

**Table S5.** Concordant overlap of differentially expressed genes and proteins resulting from transcriptomic and proteomic analyses of HT29 Luc vs. CD44 kd xenograft tumors.

**Table S6.** Oxidative phosphorylation (OxPhos) genes differentially expressed between HT29 Luc vs. CD44 kd xenograft tumors.

## Data Availability

RNA sequencing data of xenograft samples are available in Gene Expression Omnibus (https://www.ncbi.nlm.nih.gov/geo) under the GSE208310 accession number.
