# Supplementary figures and images for: Spontaneous metastasis xenograft models link CD44 isoform 4 to angiogenesis, hypoxia, EMT and mitochondria‐related pathways in colorectal cancer
Source: Mol Oncol. 2023 Nov 3;18(1):62–90. doi: 10.1002/1878-0261.13535 (PMC10766209; doi:10.1002/1878-0261.13535)

**A**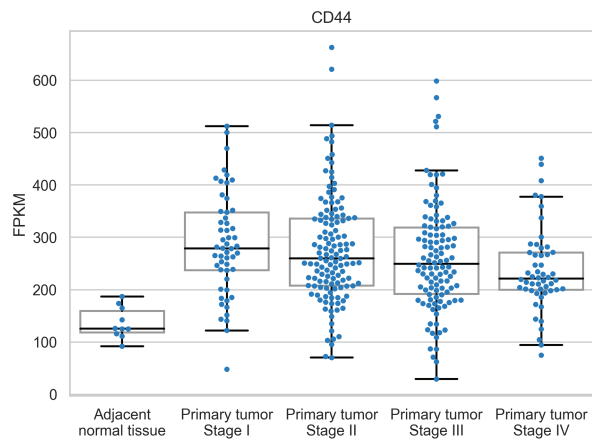**B**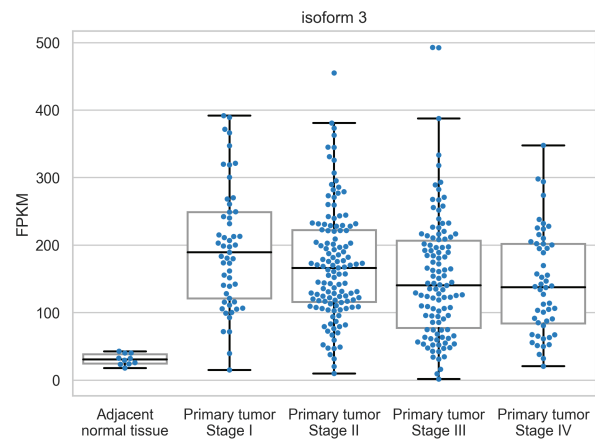**C**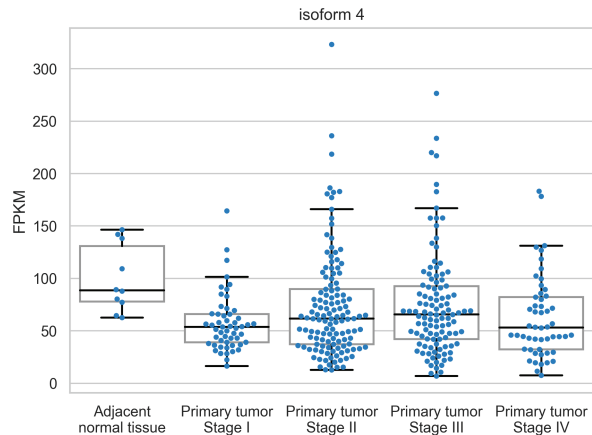**D**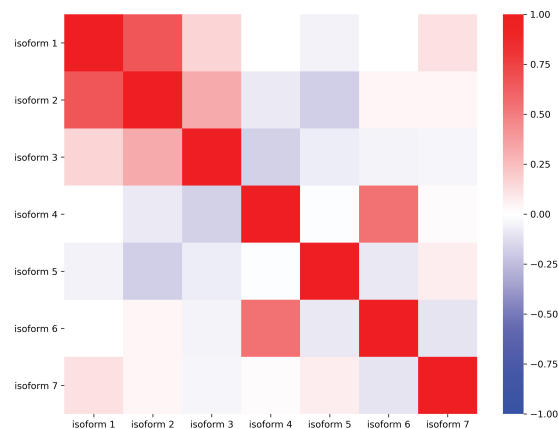

Supplement: Supplementary file 1 — Fig. S1. Dependence of CD44 isoforms' expression levels on colon cancer stage and isoform‐isoform correlations (TCGA). [file MOL2-18-62-s003.pdf]

Isoforms FPKM distribution

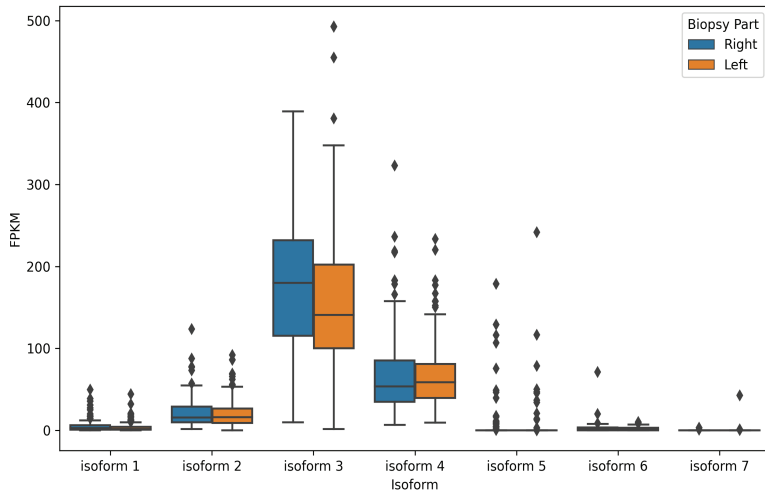

Supplement: Supplementary file 2 — Fig. S2. CD44 isoforms 1–7 expression in left and right‐sided colon cancer tissues (TCGA). [file MOL2-18-62-s009.pdf]

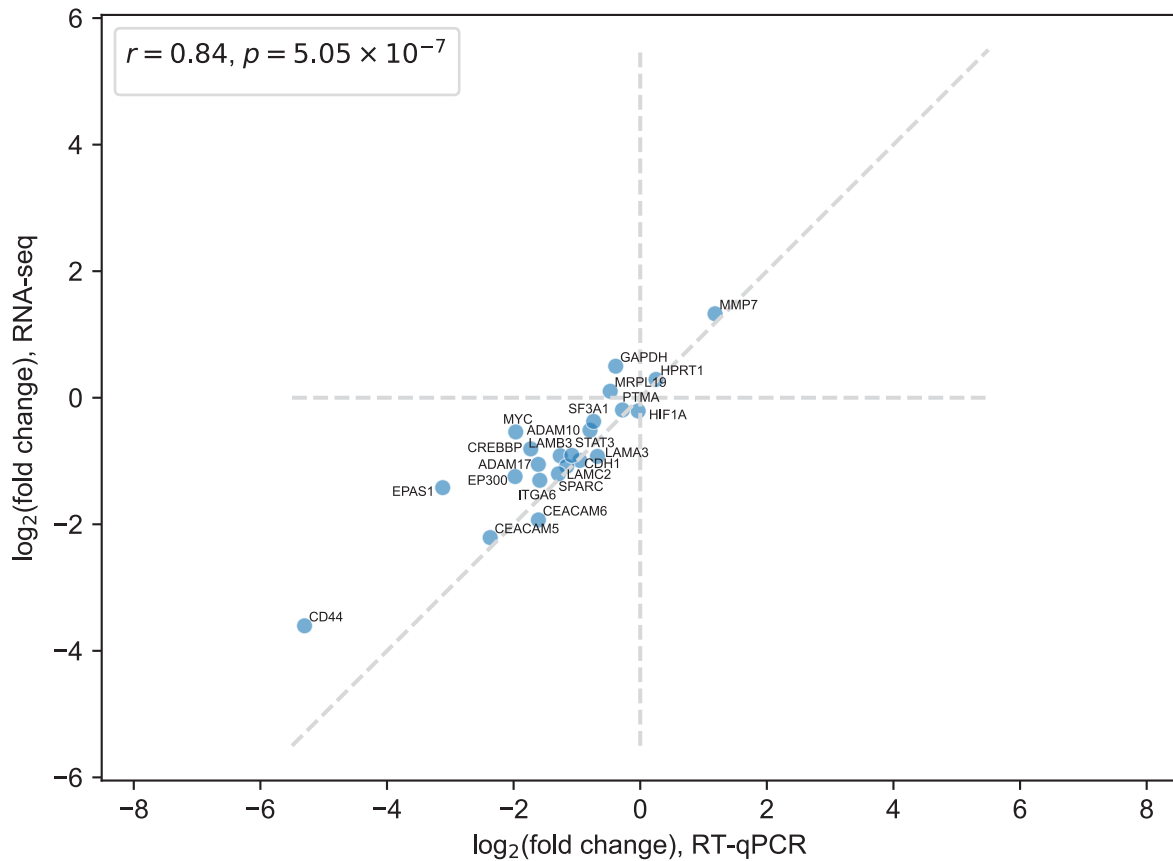

Supplement: Supplementary file 3 — Fig. S3. Validation of RNA‐Seq data by RT‐qPCR. [file MOL2-18-62-s006.pdf]

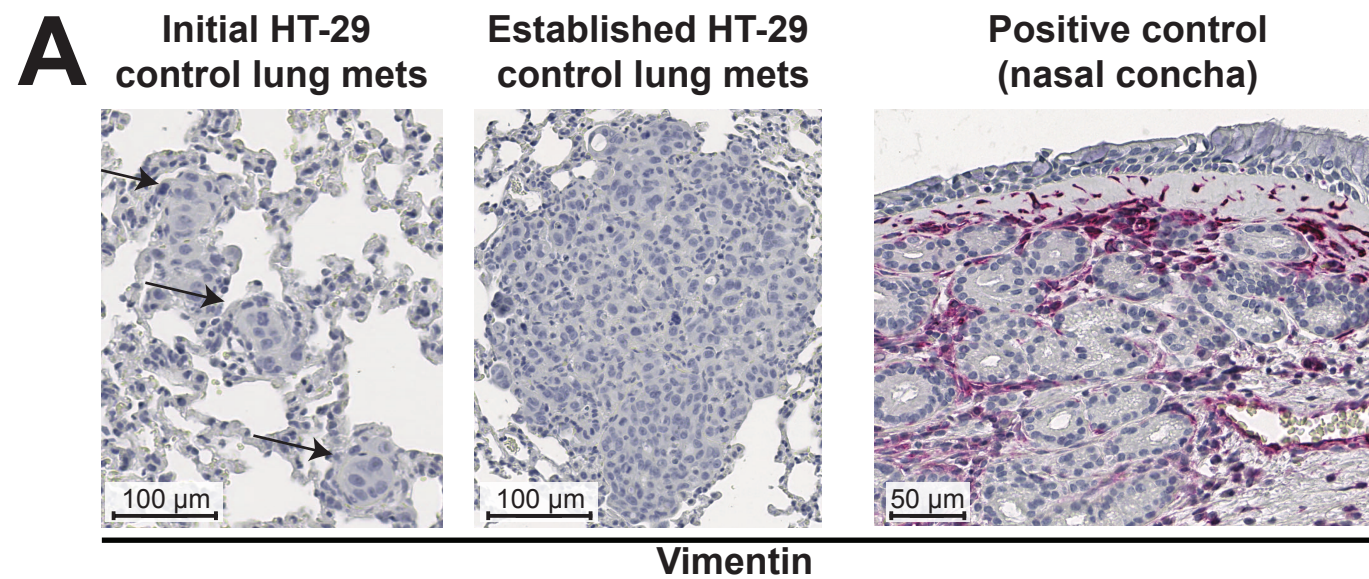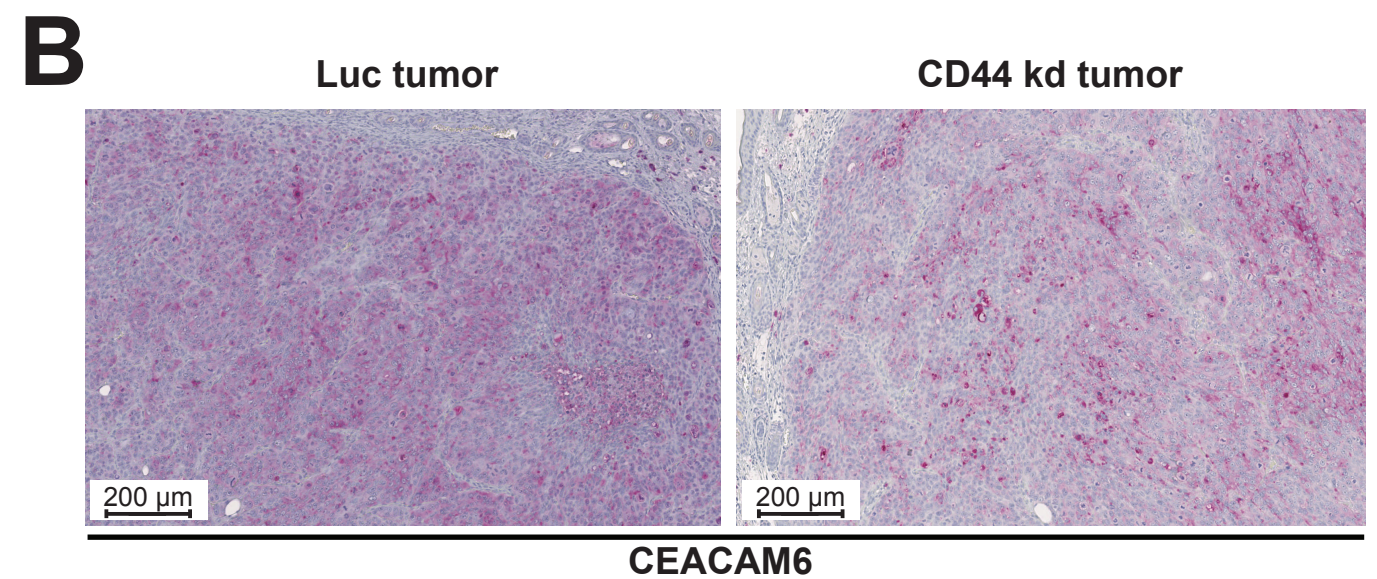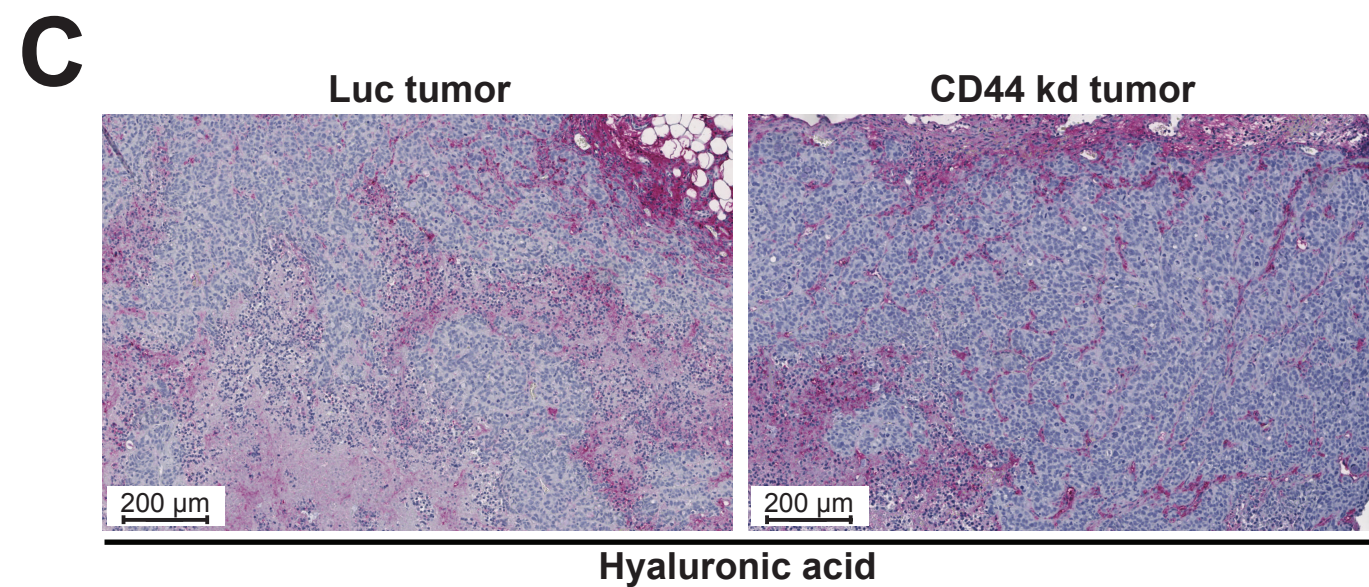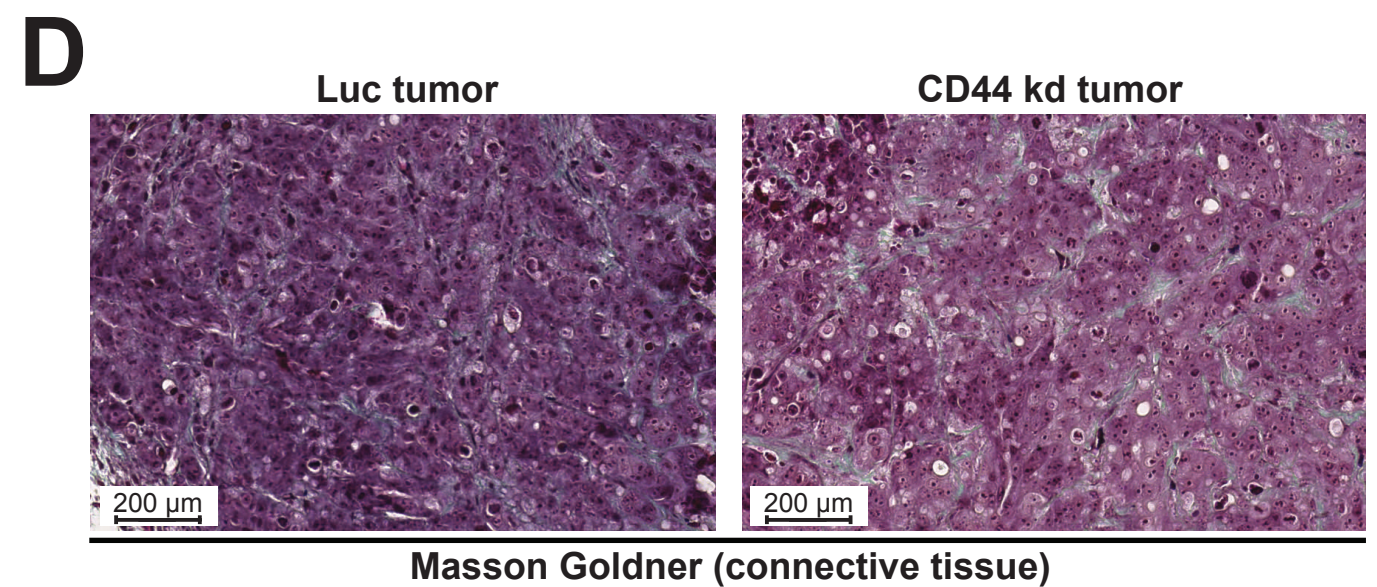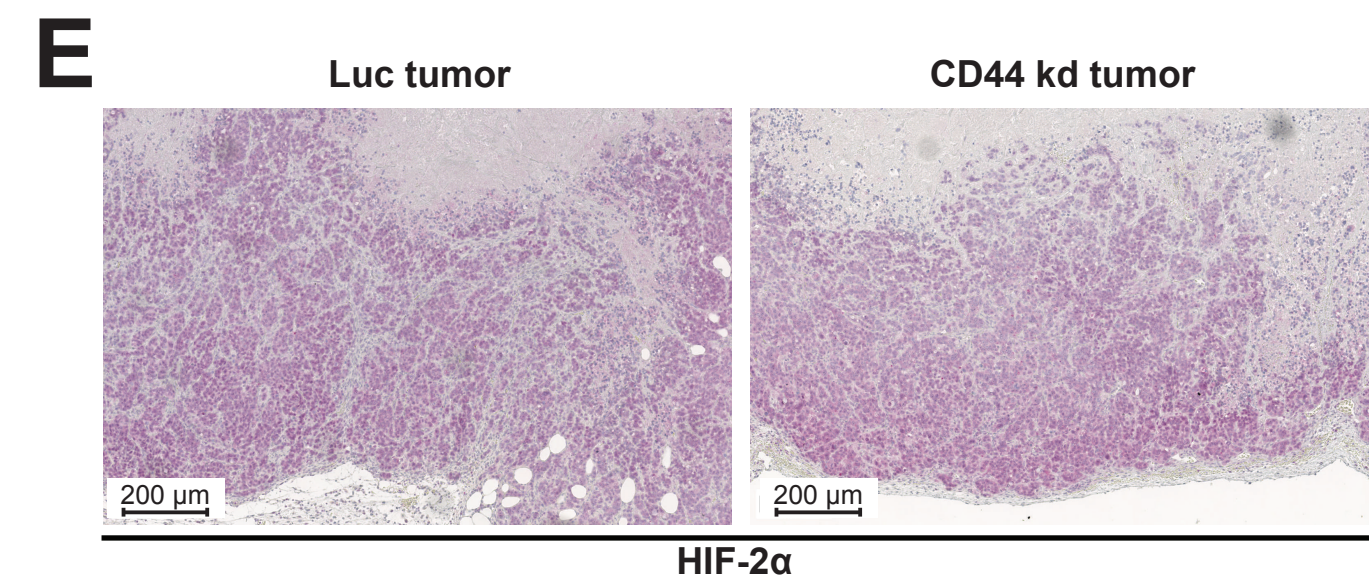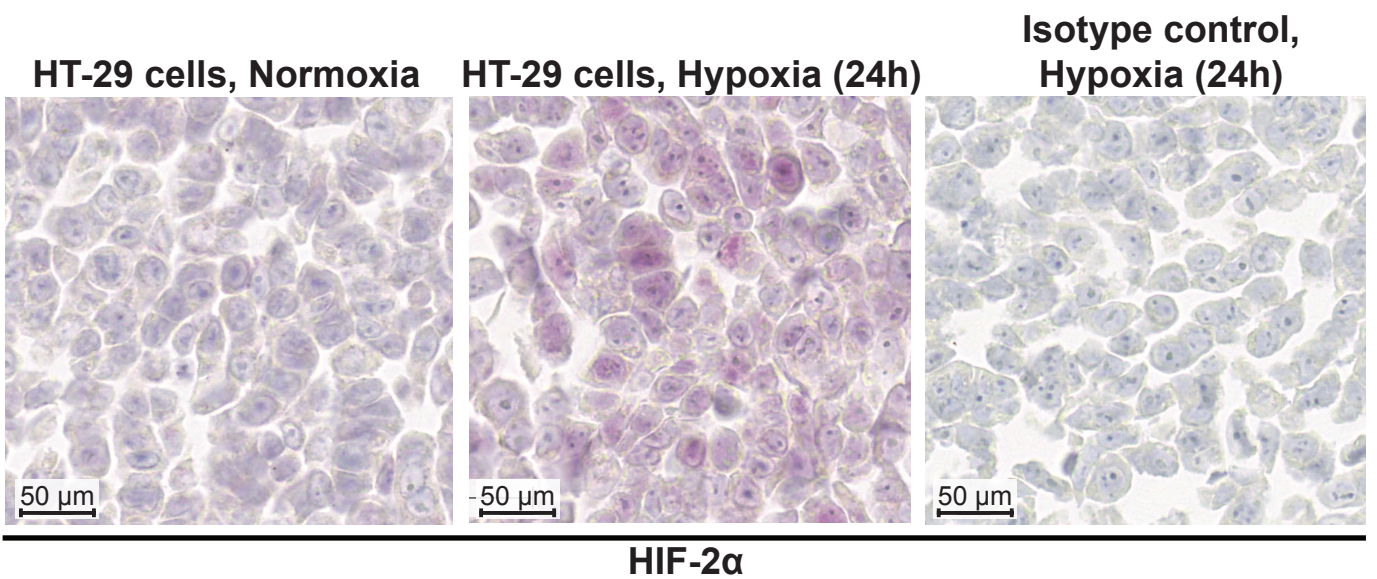

Supplement: Supplementary file 4 — Fig. S4. Expression of selected proteins in HT‐29 xenograft primary tumors, lung metastases and in HT‐29 cells under normoxic vs. hypoxic conditions. [file MOL2-18-62-s010.pdf]
