# Supplementary material for: Spontaneous metastasis xenograft models link CD44 isoform 4 to angiogenesis, hypoxia, EMT and mitochondria‐related pathways in colorectal cancer
Source: Mol Oncol. 2023 Nov 3;18(1):62–90. doi: 10.1002/1878-0261.13535 (PMC10766209; doi:10.1002/1878-0261.13535)
Supplement: Supplementary file 5 — Fig. S5. 2D cell culture‐based in vitro assays for tumor growth‐ and metastasis‐related tumor cell features. [file MOL2-18-62-s005.pdf]

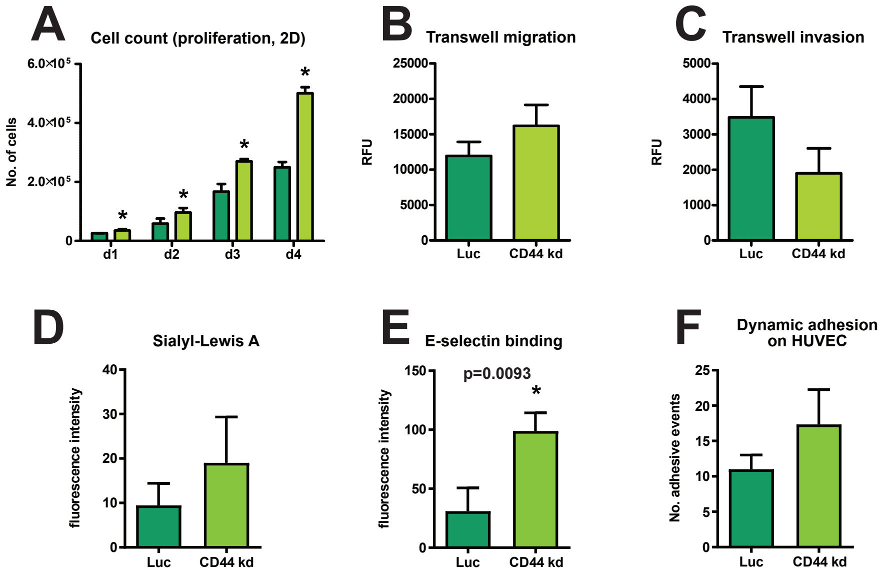

A
Cell count (proliferation, 2D)
No. of cells
6.0×10^5
4.0×10^5
2.0×10^5
0
d1
d2
d3
d4
*
B
Transwell migration
RFU
25000
20000
15000
10000
5000
0
Luc
CD44 kd
C
Transwell invasion
RFU
5000
4000
3000
2000
1000
0
Luc
CD44 kd
D
Sialyl-Lewis A
fluorescence intensity
40
30
20
10
0
Luc
CD44 kd
E
E-selectin binding
fluorescence intensity
p=0.0093
*
150
100
50
0
Luc
CD44 kd
F
Dynamic adhesion
on HUVEC
No. adhesive events
25
20
15
10
5
0
Luc
CD44 kd
