# Supplementary material for: Spontaneous metastasis xenograft models link CD44 isoform 4 to angiogenesis, hypoxia, EMT and mitochondria‐related pathways in colorectal cancer
Source: Mol Oncol. 2023 Nov 3;18(1):62–90. doi: 10.1002/1878-0261.13535 (PMC10766209; doi:10.1002/1878-0261.13535)
Supplement: Supplementary file 6 — Table S1. Summary of immunohistochemical staining protocols relevant to this study. [file MOL2-18-62-s007.docx]

**Table S1.** **Summary of immunohistochemical staining protocols relevant to this study.**

| Target (clone) | Antigen retrieval | Primary antibody (company#, working conc.) | Isotype control | Secondary antibody | Detection |
| --- | --- | --- | --- | --- | --- |
| Pan-CD44  (C26, G44-26) | Citrate buffer pH 6.0  water bath 85°C, 10 min | BD Pharmingen #550392,  25 µg/ml | Mouse IgG2b | Goat anti-mouse biotin | Vectastain^®^ APC-AP Kit  + Permanent Red |
| CD44v9 (RV3) | Citrate buffer pH 6.0  water bath 95°C, 40 min | Biolegend  #934402,  10 µg/ml | Rat IgG2a | Rabbit anti-rat biotin | Vectastain^®^ APC-AP Kit  + Permanent Red |
| CD44v6 (CD44v6/1246) | Tris-EDTA pH 9.0  water bath 95°C, 45 min | Novus  #NBP-53203,  1 µg/ml | Mouse IgG2a | Goat anti-mouse biotin | Vectastain^®^ APC-AP Kit  + Permanent Red |
| E-cadherin  (NCH-38) | Target retrieval solution pH 6.0 (Dako)  pressure cooker 121°C, 10 min | Dako  #M3612,  0.67 µg/ml | Mouse IgG1 | Anti-mouse biotin | Dako REAL^TM^ Detection System, AP/RED, Rabbit/Mouse |
| Vimentin  (Vim 3B4) | Tris-EDTA pH 9.0  water bath 85°C, 20 min | Dako  #M7020,  0.26 µg/ml | Mouse IgG2a | Goat anti-mouse biotin | Vectastain^®^ APC-AP Kit  + Permanent Red |
| CEACAM5  (C66/1009) | Target retrieval solution pH 6.0 (Dako)  pressure cooker 100°C, 20 min | LSBio  #LS-B16921,  10 µg/ml | Mouse IgG2a | Goat anti-mouse biotin | Vectastain^®^ APC-AP Kit  + Permanent Red |
| CEACAM6  (A1E2) | EDTA pH 8.0  pressure cooker 100°C, 20 min | Thermo Fisher  #MA5-37801,  40 µg/ml | Mouse IgG2b | Goat anti-mouse biotin | Vectastain^®^ APC-AP Kit  + Permanent Red |
| mCD31  (SZ31) | Citrate buffer pH 6.0  pressure cooker 100°C, 20 min | Dianova  #DIA-310,  10 µg/ml | Rat IgG2a | Rabbit anti-rat biotin | Vectastain^®^ APC-AP Kit  + Permanent Red |
| HIF-1α  (EP1215Y) | Tris-EDTA pH 9.0  pressure cooker 121°C, 10 min | abcam  #ab51608,  7.5 µg/ml | Rabbit IgG | Goat anti-rabbit biotin | Vectastain^®^ APC-AP Kit  + Permanent Red |
| HIF-2α  (polyclonal) | Tris-EDTA pH 9.0  pressure cooker 121°C, 10 min | abcam  #ab109616,  2 µg/ml | Rabbit IgG | Goat anti-rabbit biotin | Vectastain^®^ APC-AP Kit  + Permanent Red |
| HSP60  (polyclonal) | Target retrieval solution pH 6.0 (Dako)  pressure cooker 100°C, 20 min | Novus  #NBP1-77397,  5 µg/ml | Rabbit IgG | Goat anti-rabbit biotin | Vectastain^®^ APC-AP Kit  + Permanent Red |
